# Nanoscale Metal−Organic Frameworks and Their Nanomedicine Applications

**DOI:** 10.3389/fchem.2021.834171

**Published:** 2022-01-24

**Authors:** Dan Zhao, Wang Zhang, Zhi-Han Wu, Hui Xu

**Affiliations:** ^1^ School of Marine Sciences, Ningbo University, Ningbo, China; ^2^ College of Plant Protection, Northwest A&F University, Yangling, China

**Keywords:** nanoscale MOFs, drug carriers, nanomedicine, biosensing and imaging, therapeutic preparation

## Abstract

Abundant connectivity among organic ligands and inorganic metal ions makes the physical and chemical characters of metal-organic frameworks (MOFs) could be precisely devised and modulated for specific applications. Especially nanoscale MOFs (NMOFs), a unique family of hybrid nanomaterials, with merits of holding the nature as the mainstay MOFs and demonstrating particle size in nanoscale range which enable them prospect platform in clinic. Adjustability of composition and structure allows NMOFs with different constituents, shapes, and characteristics. Oriented frameworks and highly porous provide enough space for packing therapeutic cargoes and various imaging agents efficiently. Moreover, the relatively labile metal-ligand bonds make NMOFs biodegradable in nature. So far, as a significant class of biomedically relevant nanomaterials, NMOFs have been explored as drug carriers, therapeutic preparation, and biosensing and imaging preparation owing to their high porosity, multifunctionality, and biocompatibility. This review provides up-to-date developments of NMOFs in biomedical applications with emphasis on size control, synthetic approaches, and surfaces functionalization as well as stability, degradation, and toxicity. The outlooks and several crucial issues of this area are also discussed, with the expectation that it may help arouse widespread attention on exploring NMOFs in potential clinical applications.

## Introduction

During the past 2 decades, the merging of nanotechnology and medicine has confirmed the biomedical behaviour of a series of nanomaterials and improved some of them effectively in clinic (Banerjee et al., 2020). A large number of nanomaterials have been obtained with expansive dimension scope from several nanometers to hundreds of nanometers, and the great majority of these nanomaterials have exhibited bright prospects in clinic, for example drug delivery, photodynamic therapy, thermal therapy, chemotherapy, and biomedical sensing and imaging (Coughlan et al., 2017; Yang B et al., 2019; Mir et al., 2020; Wen et al., 2021).

As a burgeoning and fascinating stream of organic–inorganic hybrid materials, Metal-organic frameworks (MOFs) are consist of inorganic metal ions/clusters and multipoint organic bridging linkers (Li S Y et al., 2017). In light of the diversity of the connections among organic linkers and metal ions, the physical and chemical characters of MOFs could be rationally controlled and regulated for particular applications, especially when minimizing the size to nanoscale regime make it a foreground platform in biomedicine (Lismont et al., 2017; Wu and Yang, 2017). Nanoscale MOFs (NMOFs) stand for a special class of hybrid nanomaterials. The merits of sustaining the properties and structural various as bulk MOFs and maintaining the size at the nanometer level let NMOFs perfectly capable applying in biomedical sciences (Lu et al., 2018a; Zhou et al., 2018; Bieniek et al., 2020; Li et al., 2020). Moreover, Compared with existing nanocarriers, NMOFs possess several potential advantages which make them have a wider application prospect for biomedicine: 1) structural and compositional adjustability permitting to build NMOFs owning diverse constitution, shapes, and characteristics; 2) oriented frameworks and high porosity provide enough space for lade therapeutic agents and various imaging efficiency; and 3) weaker coordination bond make them easily to be biodegraded.

**FIGURE 1:**
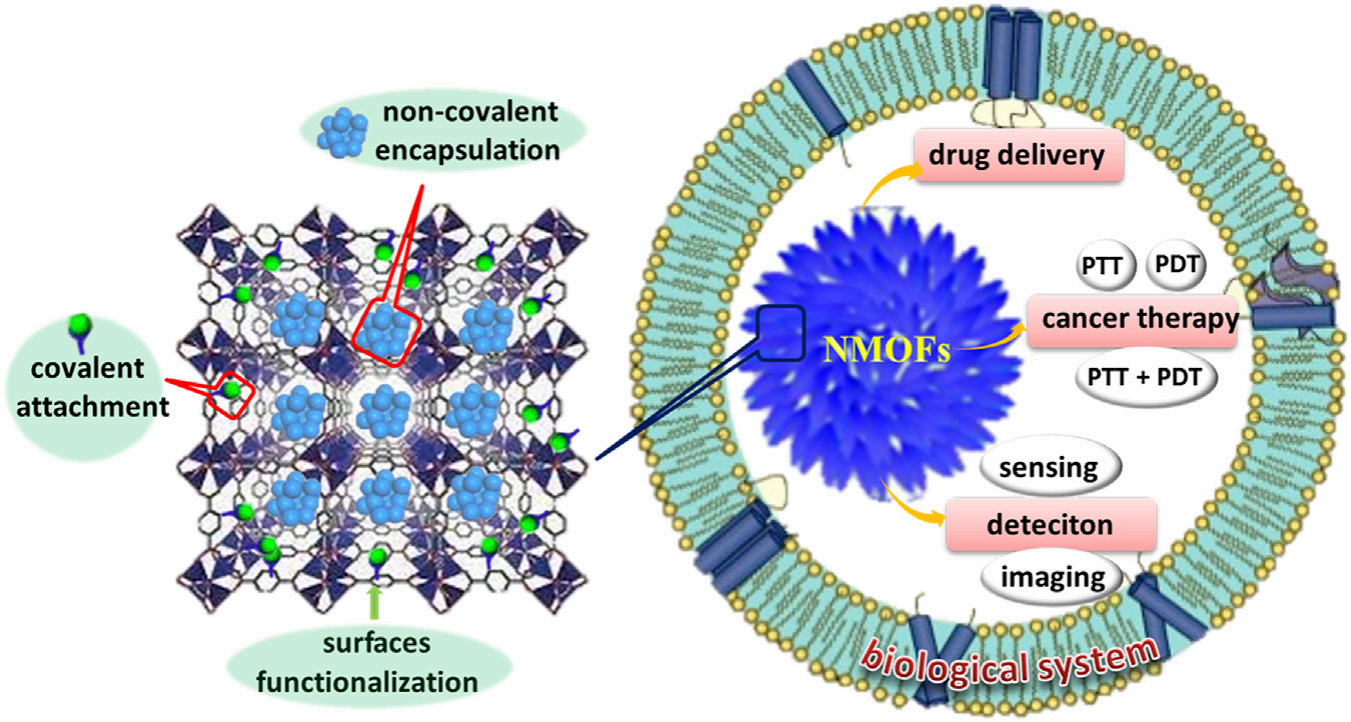
Schematic diagrams of NMOFs in biomedicine.

In this review, the main focus is to highlight the recent research progress of NMOFs in the biological area, including the development of NMOFs in targeted drug delivery and cancer therapy, biomedical sensing and imaging as well as the toxicity, degradation, and stability of this class of novel hybrid nanomaterials (Figure 1). First, the general strategies to functionalize NMOFs for biomedicine-available considerations are briefly summarized, including pore encapsulation, and surfaces functionalization. Then, recent biomedical applications of NMOFs are discussed, including NMOFs as nanocarriers for intracellular delivery of drugs; as phototherapeutic agents for cancer therapy; and as fluorescent probes for biomedical sensing and imaging. Additionally, the outlooks and several crucial issues of this area are also mentioned with the expectation of stimulating more attention on investigating the potential of NMOFs for clinical applications.

## Nanoscale Metal–Organic Frameworks

Nanoscale metal-organic frameworks (NMOFs), a peculiar class of metal-organic self-assembly materials, integrate the merits of beneficial property of bulk MOFs and nanomaterials. First, highly porous and large channels enable NMOFs loading imaging agents and/or therapeutic preparations efficiently meanwhile by covalent attachment the construction units—whether metal ions/cluster or organic ligands—both can be used to payload imaging cargoes and/or drugs (Chen D et al., 2017; Hang et al., 2017; Yin et al., 2017; Cao et al., 2021). For example, by choosing construction units or decorating imaging components, NMOFs-based diagnostic and therapeutic agents have been extensively developed and used for magnetic resonance imaging (MRI) (Chowdhuri et al., 2016), computed tomography (CT) (Tian et al., 2015), positron emission tomography (PET) (Zhao et al., 2016a), and optical imaging (Yang S P et al., 2017). Second, appropriate selection and functionalization of organic linkers and building blocks allows NMOFs to load and release imaging agents and drugs components in steerable patterns both spatially and temporally, emphasizing their latent roles on theranostic platforms (Ni et al., 2020a). The inorganic metal components on NMOFs also make them attractive contrast medium for biomedical optical applications. So far, therapeutic construction units have been encapsulated into NMOFs for particular treatment. Such as, using photosensitizers as construction groups permit the NMOFs effective for photodynamic therapy (PDT), meanwhile, high-Z metal ions-containing NMOFs materials possess excellent X-ray attenuation ability could be used as radiosensitizer to improve field radiotherapy (RT) (Nazari et al., 2016). Third, the adjustable skeleton stability of NMOFs in physical surroundings permits to obtain activated or controlled-release nanocarriers. Additionally, many sorts of NMOFs have been certificated with low cytotoxicity ensures them applications *in vivo*. Also, in light of the comparatively weaker metal-ligand coordinate bonds, the NMOFs are biodegradable in nature (Morris et al., 2017). These exceptional merits of the NMOFs make them a promising platform for biomedical field.

### Synthesis and Design Strategies of Nanoscale Metal–Organic Frameworks-Based Nanomedicine

**FIGURE 2:**
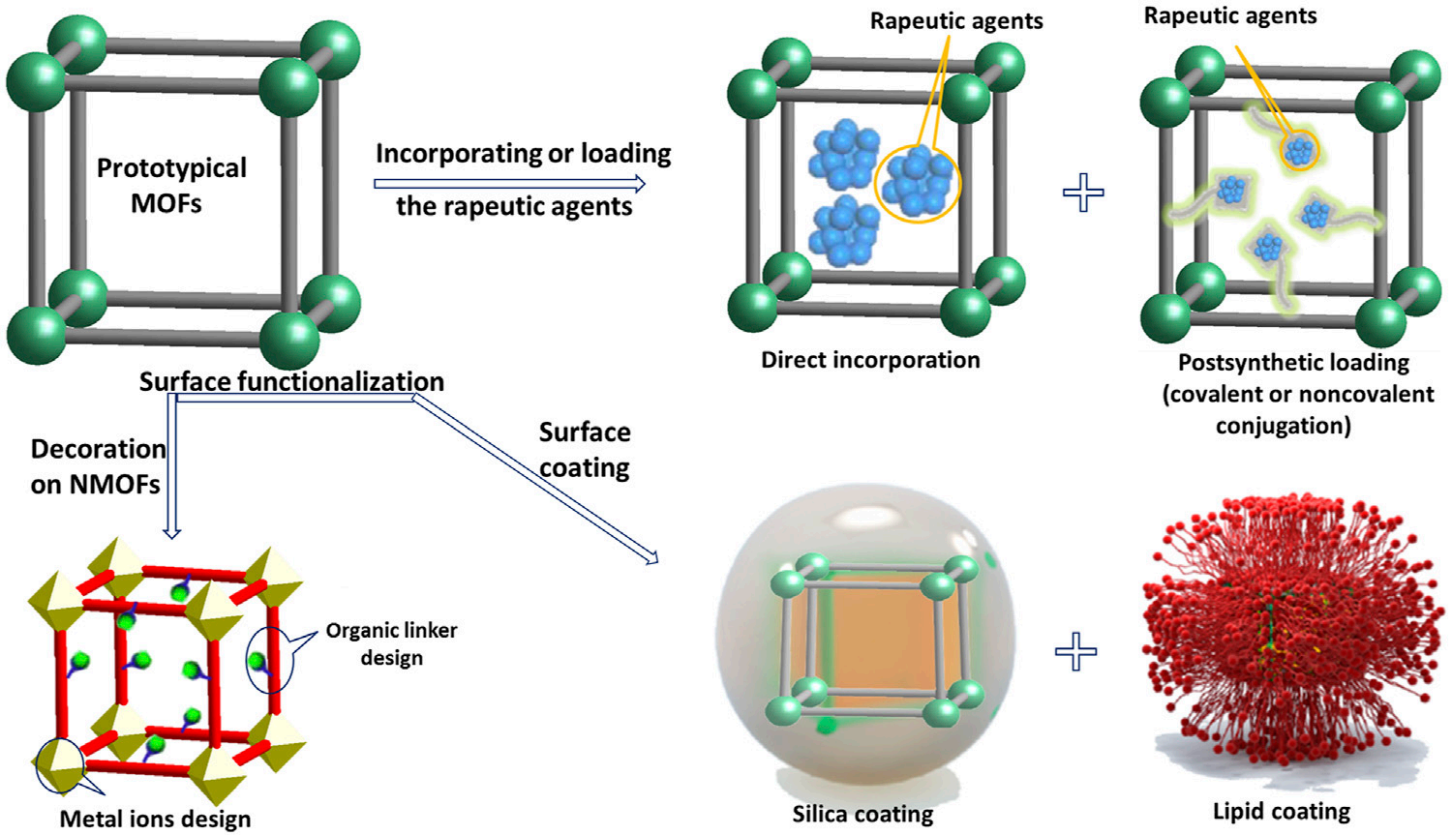
Approaches described in this article for constructing NMOFs-based nanomedicine.

For biomedical applications, the first attention should be paid to scaling down particle size within nanoscale (Cai M et al., 2020). As porous nanomaterials, NMOFs could be designed with a limitless array of inorganic and organic components. Normally, to acquire nanoscale MOFs, two factors should be considered: 1) limiting the supramolecular assembly; a process that usually results in the formation at nanoscopic locations and 2) supporting nucleation against crystal growth. Currently in literature, controlled synthetic techniques of NMOFs gradually have been divided into four categories: nanoprecipitation, solvothermal synthesis, surfactant-assisted approach, and reverse microemulsion method (Rieter et al., 2008; Liu et al., 2012; Baa et al., 2019). In these methods, reactant ratio, temperature, pH, reaction conditions, etc. factors affect the growth and nucleation of NMOFs during the synthetic progress (Zhao et al., 2018). NMOFs occupy various merits over traditional nanomedicines for example their structural diversity, their loading capacity, and their biodegradability. A great deal of NMOFs has been certified as bioimaging agents both *in vivo* and *in vitro* (Cai et al., 2015). Researchers have used two general approaches to construct NMOFs-based nanomedicine, as shown in Figure 2: one is incorporating active groups into the skeleton or loading active agents into the channels and pores of the NMOFs; the other one is surface modification of as-synthesized NMOF by silica coatings or organic polymers to improve their stabilities, slightly regulated their physicochemical characteristics, and afford extra functionality and biocompatibility. For pore encapsulation strategy: NMOFs as a host material can prevent the loaded substrates from leaching, as well as providing them a protective environment against external adverse factors. Surfaces functionalization strategy for NMOFs can meet the specific requirements and acquire the expected function, which is extremely significant in clinic. However, these two strategies require that NMOFs and the substrate are stable under synthetic conditions.

Incorporating or loading the rapeutic agents within NMOFs: Molecular nature of NMOFs can give materials various characters. The moderate synthetic conditions and tunable components of NMOFs allow for the capacity of therapeutic and imaging agents while at the same time guaranteeing effective delivery to internal specific cells (Simon-Yarza et al., 2018). Generally, there are two general categories for cargo loading in NMOFs: incorporating cargoes directly during particle synthesis and postsynthetic loading. Additionally, complementary of these two strategies can also be applied for merging multiple biomedically relevant groups and NMOFs to develop theranostic agents.

In the method of direct incorporation, bioactive agents (the metal notes or the linkers) can be incorporated within NMOFs directly during their self-assembling process. The most commonly used therapeutic preparations based-MOFs are generally have fairly good stability in water solution such as Zr-MOF (Abánades Lázaro et al., 2020), porphyrinic MOF (Li et al., 2021), zeolitic imidazolate frameworks (ZIF) (Pan et al., 2017), and Fe-MOF (Cui et al., 2021). Although extremely high agents loading with equidistribution could be achieved by direct incorporation method, but the physic-chemical characters and morphology of these materials require to be adjusted separately. Apart from being incorporated directly, biomedically relevant units can also be imported within NMOFs in the synthesis process by object encapsulation. Various agents with different properties have been used to encapsulate into NMOFs by this strategy for example proteins, small particles, dyes, and drugs ^[35]^. But, this method can only achieve a lower drug loading, and that the release kinetics of the drug from the NMOF and the skeleton decomposition rate may be extraordinary different.

Postsynthetic loading is another loading method for encapsulating biomedically relevant molecules within NMOFs. After obtaining highly porous NMOFs with specific physiochemical properties, the active units are then packed into the NMOFs via covalent or noncovalent surface conjugation. Covalent postsynthetic modification is realized by functional groups in NMOFs react with bioactive reagents (Nguyen et al., 2010). As a case, a BDC-NH_2_ (2-amino terephthalic acid) group including NMOFs has been used to link contrast medium and chemotherapeutic agents via covalent conjugation (Huang et al., 2006). Noncovalent surface conjugation offers another effective way for loading drugs and an extraordinary load amount with 1.4 g of ibuprofen/g was achieved in MOF-101 (Horcajada et al., 2006).

Surfaces functionalization of NMOFs: Surfaces functionalization of NMOFs to meet the specific requirements and acquire the expected function is extremely significant in clinic. The controlled manipulation of the external surface of NMOFs improves the water dispersibility, reduces nonspecific binding with plasma protein, and appends other components to specific areas or to add extra functionalities or groups (Horcajada et al., 2006; Alexis et al., 2008).

Since NMOFs are formed by ligands and ions, the primary task is to decorate a connector on ligands before MOF assembly and then covalently bonding the specific agent with the connector on the superficies of the as-synthesis NMOFs (Zhu et al., 2018). For example, as the organic ligand in UiO-66, 1,4-benzenedicarboxylic acid without connector lead to the resultant UiO-66 cannot be functionalized by covalent modification. Conversely, 2-azido terephthalic acid, the organic linkers of UiO-66-N_3_, where the azide group could react with the alkane group by click reactions to modify the desired agent on the surface of MOF. Finally, a dibenzylcyclooctyne terminal DNA sequence sucessful bonded with the surface of UiO-66-N_3_ without any internal change in the whole chemical transformation process (Morris et al., 2014). The second strategy is using the chelating ability of metal ions and target molecule to connect the specific agents on the MOF surface directly (Wang et al., 2017; Wang et al., 2019). Surface defects of NMOFs, such as the unsaturated coordinative metal sites (CUS), give NMOFs the ability to combine with target molecules by coordination. At present, different functional group-terminal ligands have been incorporated to NMOFs by coordinating to the metal-sites through external secondary modification including phosphonate, carboxylate, phenyl, and histidine groups. For example, both phosphonate and carboxylate groups can bonding with Zr ions in Zr-based MOFs, thus decorating the surface of the Zr-based MOF with terminal phosphate-modified DNA successfully (Röder et al., 2017). His-tags can be easily fabricated in peptides or proteins greatly expanding the range of targeted molecule-functionalized MOFs. Phenolic capped linkers also can generate the coordination bonds to modify MOF a stable material via post-synthetic (covalent) modification (Wang et al., 2015). A great variety of ions have been certified to be able to chelate with phenolic units to afford stable 5-member ring including Fe, Zr, Cr, Cu, Zn, Co, In, Eu, and Al.

Surface coating provides another convenient way to modify NMOFs with their structural integrity and porosity unchanged. During surface coating process, some factors should be considered: 1) biofriendly synthetise without any poisonous substance; 2) prevent the targeting molecule invading into the nanocarrier; 3) reduce interference with entrapped drugs; 4) keep drug release capacity; 5) improve the stability of particle colloidal, and 6) stable under physiological environment. So far, silica coating and lipid coatings have been considered the most effective strategies for NMOFs surface coating, especially for stabilizing physiologically unstable NMOFs. For promoting process of the silica coating, firstly the surface of NMOFs is decorated with a polymer, like polyvinylpyrrolidone (PVP). For instance, Lin and co-workers have obtained a series of NMOFs by silica coating (Rieter et al., 2008). Authors represented an universal way to acquire different silica shells thickness on the surface of Ln (BDC)_1.5_(H_2_O)_2_ (Ln = Gd^3+^, Eu^3+^, or Tb^3+^, BDC^2-^ = 1,4-benzenedicarboxylate); Tb_2_ (DSCP)_3_(H_2_O)_12_ (DSCP = c,c,t-(diamminedichlorodisuccinato), Mn_3_(BTC)_2_(H_2_O)_6_ (BTC^3-^ = 1,3,5-trimesic acid), and Cr_3_F(H_2_O)_2_O(BDC)_3_·*n*H_2_O (where *n* is ∼25). Eventually, they loaded dipicolinic acid (DPA), Pt-based drugs, acyclicarginine-glycine-aspartate (RGD) peptide, and optical imaging contrast agent (BODIPY) into nanoscale materials stabilized with silica shell for biological application. Although silica coating method seems to be a widespread approach for various materials, it is hard to gain flimsy silica shells. Furthermore, weakly basic coating condition is incompatible with material. Lipid coating is not only available for slowing down the kinetics of releasing cargos but also efficient for imparting biocompatibility (Wuttke et al., 2015). The Mirkin group used the lipid ligand 1,2-dioleoyl-snglycero-3-phosphate (DOPA) to transfer a family of Zr^IV^-based NMOFs facilely be dispersed and suspended in solvents with low polarity by easily surface encapsulation. Differ from artificial lipid layers, exosomes which have membrane structures similar with cell membranes, are usually used by cells for communication purposes. The Wuttke and co-workers coated exosomes on MIL-88A surface successfully to investigate the sustained-release of calcein in HeLa cells (Illes et al., 2017). Although a significant progress have been achieved in NMOFs modification, there are still unsolved problems in aspect of concrete characterization of targeting/coating stability and efficiency (Meng et al., 2016; Abánades Lázaro et al., 2017). In addition, for fulfilled potential of NMOF-based therapeutic and imaging agents, rigorous *in vivo* and *in vitro* researches are acquired.

## Nanomedicine Applications of Nanoscale Metal–Organic Frameworks

Unique molecular architecture and versatile functionalities make NMOFs potential platforms for drug delivery, cancer therapy, biomedical sensing, and imaging applications (Velásquez-Hernández et al., 2020). In this section, the parameters of NMOFs before the biomedical applications of NMOFs will be discussed and the recent research progress related to nanomedicine applications of NMOFs in clinical is summarized in the context of translational medical research.

### Parameters of Nanoscale Metal–Organic Frameworks in Biomedicine

Although most NMOFs have the characteristics of high surface areas, tunable pore size, crystallinity, thermal stability, and so on, but for medical applications, some strict parameters should be considered (McKinlay et al., 2010). Once MOFs are utilized in biologically and medical applications, characteristics like stability, biodegradability, and toxicity need to be addressed further.

Stability: Before the successful applications of NMOFs in diagnosis and treatment, the stability in the physiological surrounding of NMOFs should be discussed. By scanning electron microscope (SEM), high-resolution transmission electronic microscope (HRTEM), X-ray diffraction (XRD), and other experimental characterization methods, the stability of NMOFs in water solution, buffer solution, and different pH conditions could be evaluated. Although few NMOFs are obtained from the aqueous phase, most of them are still stable in the aqueous solution, including UiO MOF series, PCN MOF series, Ni-CPO-27, Cr-MIL-100, Cr-MIL-101, MIL-125, CAU-1, and ZIF-8 (Yang et al., 2011; Jeazet et al., 2012; Liu et al., 2015). Lin groups obtained a nanosensor on account of fluorescein isothiocyanate (FITC) conjugated UiO-68 (F-UiO) for pH sensing in cell (He et al., 2014a). The fluorescein FITC is pH sensitive and shows pH-dependent ratiometric fluorescence changes, certificating its ability to sense the pH_i_ in cells. F-UiO exhibited unaltered hexagonal plate morphology after incubating in Hank’s Balanced Salt Solution (HBSS) for 24 h. Negligible FITC dye leaching experiment and XRD patterns indicate that the structural of F-UiO was stable in cell.

Degradation: Biodegradability of MOFs is another important issue that needs to be emphasized in term of biological application (Wu et al., 2018). By compared the release mechanism of drug in NMOFs with two other coating methods, Amoriń-Ferré and co-workers found that NMOFs with a formula of [Co(bix)(3,5-dbsq)(3,5-dbcat)] (bix = 1,4-bis(imidazol-1-ylmethyl)benzene) could be designed in spherical structures with a diameter of 150–200 nm, where 3,5-dbsq^-^ and 3,5-dbcat^2-^ are respectively the semiquinonate radical and catecholate forms of 3,5-di-tert-butyl-1,2-benzoquinone (dbq) (Amoriń-Ferré et al., 2013). Physical encapsulated drugs within NMOFs will be released by free diffusion, but covalently bound drugs within the structures can only be governed by slow particle degradation. Detailed description of this situation in the case of MIL-100(Fe) nano- or microparticles confirmed the gradual formation of dense forms in the process of degradation in a simulated physiological environment. Also, the excretion and degradation mechanism proved that the Fe-MOFs could be quickly segregated by organism and then eliminated and biodegraded safely.

Toxicity: For the ultimate biomedical goal of NMOFs in clinical, toxicity issues should be solved by a more reasonable safe-by-design approach. As MOFs consist of metal ions/cluster and organic linkers, some nontoxic or low-toxic inorganic metal ions and organic ligands should be selected to produce NMOFs for reducing of toxicity from the source. As kinds of essential microelements to human body, Cu, Zn, Fe, Mn, Mg and Zr metal ions, have been heavily applied in NMOFs assembly with acceptable toxicity decided by oral lethal dose 50 (LD50), i.e., 350 μg kg^−1^ for Zn, 25 g kg^−1^ for Cu, 30 g kg^−1^ for Fe, 4.1 g kg^−1^ for Zr, and 1.5 g kg^−1^ for Mn (Amoriń-Ferré et al., 2013; Wu and Yang, 2017). As for exogenous organic linkers, terephthalic acid (LD50 = 5 g kg^−1^), fumaric acid (LD50 = 10.7 g kg^−1^), trimesic acid (LD50 = 8.4 g kg^−1^), terephthalic acid (LD50 = 5 g kg^−1^), 5-aminoisophthalic acid (LD50 = 1.6 g kg^−1^), 2,6-napthalenedicarboxylic acid (LD50 = 5 g kg^−1^), isonicotinic acid (LD50 = 5 g kg^−1^), 1-methylimidazole (LD50 = 1.13 g kg^−1^), and 2-methylimidazole (LD50 = 1.4 g kg^−1^), are normally significant for bioapplications. Horcajada and co-workers did outstanding works on screening the general toxicity and cytotoxicity of a range of NMOFs in mice (Tamames-Tabar et al., 2014). In their experiment, three Fe-based NMOFs could be rapidly absorbed by mononuclear phagocytic system organs after intravenous injection. The Maspoch group systematically described *in vivo* toxicities of a series of NMOFs such as MIL-88A, MIL-88B-4CH_3_, MIL-100, MIL-101, NOTT-100, HKUST-1, ZIF-7, ZIF-8, MOF-74 family (M = Ni, Co, Cu, Mg, Zn, and Mn), UiO-66, UiO-66-NH_2_, UiO-67, and MOF-5 (Ruyra et al., 2015). The authors found that NMOF toxicity has a strong correlation with metal toxicity as well as particles shape/size**.**

### Synthesis and Design Strategies of Nanoscale Metal–Organic Frameworks-Based Nanomedicine

Drug delivery have appeared as an important field of research due to the unacceptable fact is that traditional delivery patterns usually refer to large doses resulting in toxic side-effects by systemic circulation at unexpected targets (Li W et al., 2017). As efficient drug delivery vehicle, some requirements should be addressed, such as 1) controlled release the active agents; 2) maximum degradation and possibility to be surface modified by the delivery vehicle; 3) could be detected by diverse imaging techniques; 4) with high drug loading capacity; 5) toxicity and biocompatibility. The merits of diversified structural components and abundant performance make NMOFs have higher drugs loading ability, controlled release ability, and are easily modified by active agents (Al Haydar et al., 2017). Another aspect that has proved MOFs more attractive as drug delivery carriers is that a sole MOF can be applied to load multiple active agents as some of these materials exhibit a dual hydrophilic/hydrophobic pore structure. Table 1 summarizes the drug release ability of various NMOFs as drug carriers. In NMOFs as carriers for targeted drug delivery system, two factors involve loading efficiency and stimulus-responsive drugs release should be discussed.

**TABLE 1:** The drug release ability of various NMOFs as drug carriers.

NMOFs	Drugs	Drugs loading effective (wt%)	Ways to stimulate drug release	Refs
MIL-53	ATP-TP; Bu; CDV; Doxorubicin (doxo)	0.24 of AZT-TP, 14.3 of Bu	-	Horcajada et al. (2010)
MIL-88A		0.6 of AZT-TP, 8.0 of Bu, 2.6 of AZT-TP, 8.0 of CDV		
MIL-89		9.8 of Bu, 14 of CDV		
MIL-100		25 of Bu, 21 of AZT-TP, 16 of CDV, 29 of doxo		
MIL-100-NH_3_		42 of AZT-TP, 41.9 of CDV		
UIO-MOFs	cisplatin	12	-	He et al. (2014a)
Se/Ru@MIL-101	siRNAs	13.93	-	Chen D et al. (2017)
Fe-MIL-88B-NH_2_	1,4,7-triazacyclononane-1,4,7-triacetic acid (NOTA) 5-amino-3-(pyrrolo [2,3-c]pyridin-1-yl)isoquinoline (defluorinated MK6240, DMK6240) methylene blue (MB)	15 of MB	-	Zhao et al. (2020)
ZIF-8	DOX	15	pH	Zheng et al. (2016)
	3-MA	19.798	pH	Chen W H et al. (2018)
	glucose oxidase (GOx), insulin	15.6 of GOx, 52.3 of insulin	ROS, Glucose, pH	Lu K et al. (2016)
NU-1000	insulin	40	-	Chen W H et al. (2018)
NU-1000x (x = 3, 4, 5, 6, 7)	Lactate dehydrogenase (LDH)	-	-	Li P et al. (2018)
ZIF-90	Rhodamine B (RhB)	-	ATP-responsed	Deng et al. (2017)
	sgRNA), CRISPR/Cas9	-	ATP-responsed	Yang X et al. (2019)
ZJU-64	MTX	13.45	Temperature, pH	Lin et al. (2016)
ZJU-64-CH_3_	MTX	10.63	Temperature, pH	Lin et al. (2016)
UIO-66-NH_2_	5-fluorouracil (Fu)	-	Temperature, Zn^2+^	Tian et al. (2015)
Bio-MOF-1	Procainamide	-	-	An et al. (2009)
MIL-101	BODIPY	5.6–11.6	-	Taylor-Pashow et al. (2009)
Zr-based-MOF	diclofenac sodium (DS)	58.80	Pressure, 37°C	Jiang et al. (2016)

Loading efficiency: Thanks to their porous structure, NMOFs have a more drug loading efficiency compared to conventional materials. The loading agents can range from small molecules to proteins. Perfect NMOF therapeutic preparations typically have larger pore size and excellent aqueous stability, such as Zr-MOF series, ZIF series, porphyrinic MOF series, and Fe-MOF series larger pore size and excellent aqueous stability (Cai et al., 2019; He et al., 2019; Abánades Lázaro and Forgan, 2019; Cai X et al., 2020; Sun et al., 2020). Horcajada groups demonstrated a family of non-toxic iron (III) carboxylate NMOFs as degradable drug carriers to transport various drugs that are hard to deliver by other nanocarriers, such as MIL-53, MIL-88A, MIL-88Bt, MIL-89, MIL-100 and MIL-101-NH_2_ (Horcajada et al., 2010). As drug carriers, their loading efficiency was detected by four commonly anticancer or antiviral drugs (azidothymidine triphosphate (AZT-TP), busulfan (Bu), cidofovir (CDV) and doxorubicin (doxo)), which, except the latter, could not be successfully encapsulanted using existing nanocarriers. The drug loading experiment showed that the NMOFs serve as a remarkable molecular ‘sponges’. For example, up to 25, 21, 16 and 29 wt% of Bu, AZT-TP, CDV and doxo could be loaded into MIL-100. Moreover, a remarkable capacity of 42 wt% can be obtained for AZT-TP and CDV with MIL-101-NH_2_.

**FIGURE 3:**
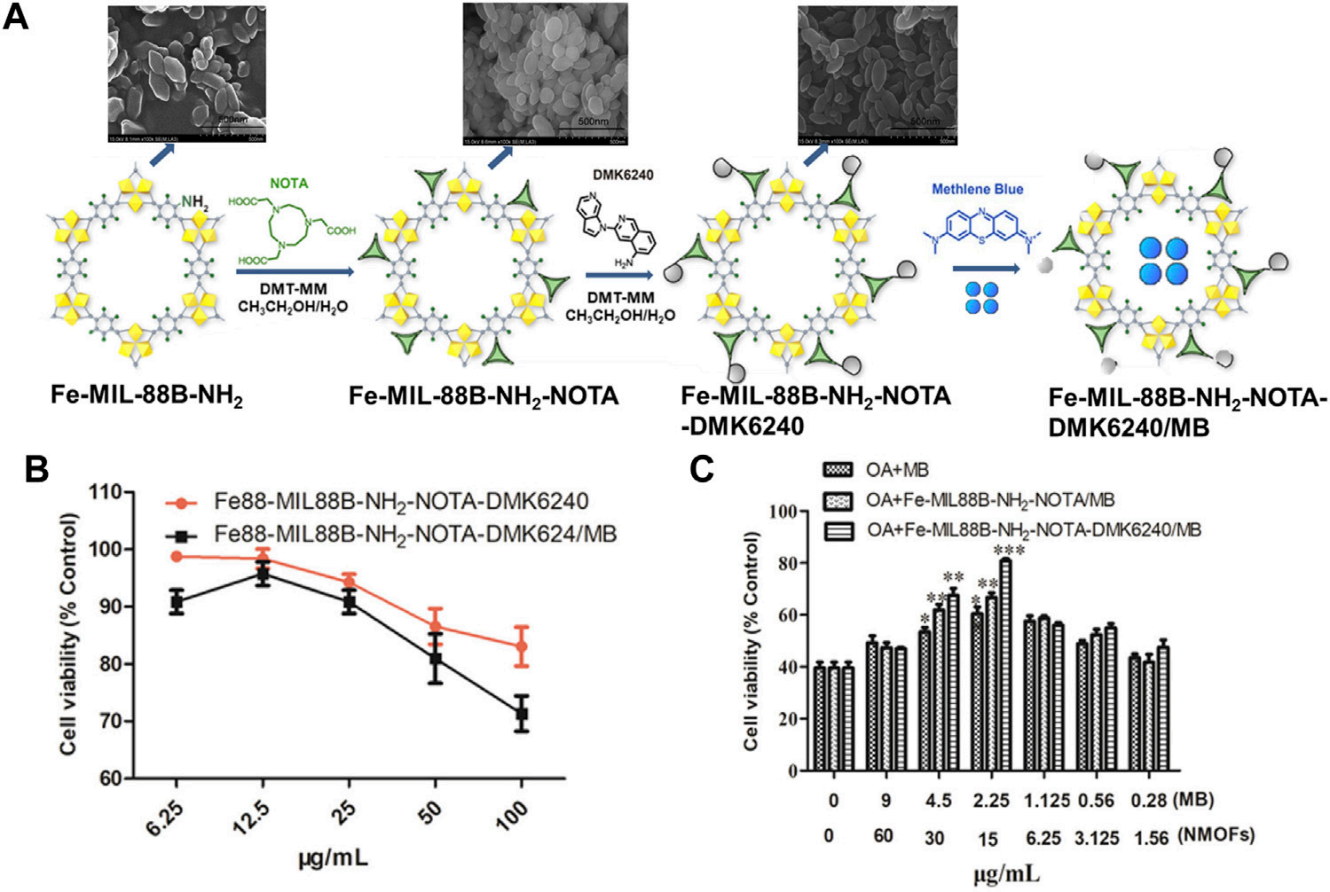
**(A)** The schematic description of the synthesis process for Fe-MIL-88B-NH_2_-NOTA-DMK6240/MB. **(B)** Viabilities of SH-SY5Y cells cultured with Fe-MIL-88B-NH_2_-NOTA-DMK6240 and Fe-MIL-88B-NH_2_-NOTA-DMK6240/MB assessed by MTT. **(C)** Cytoprotection effect of Fe-MIL-88B-NH_2_-NOTA-DMK6240/MB. The OA-induced SH-SY5Y cell was treated with MB, Fe-MIL-88B-NH_2_-NOTA/MB, and Fe-MIL-88B-NH_2_-NOTA-DMK6240/MB for an additional 12 h. Source: (Zhao et al., 2020).

Expect for single drug delivery, MOFs also can be serving as a nanocarrier for drug co-delivery to improve clinical effects. For example, Lin group successfully loaded siRNA and cisplatin together into UiO-NMOFs via encapsulation and surface coordination, respectively (He et al., 2014b). Cisplatin loading capability is up to 12 wt%. Later, Liu and co-workers decorated selenium (Se) and ruthenium (Ru) NPs and loaded siRNAs in the surface of MIL-101 successfully (Chen Q et al., 2017). The combination of Se/Ru-NMOFs and siRNA not only can significantly improve the therapy efficacy but also has high tumor-targeted, enhances antitumor efficacy, and reduces systemic toxicity *in vivo*. Recently, a multifunctional NMOF-based drug delivery system was obtained by Kong groups for targeting hyperphosphorylated tau (Zhao et al., 2020). As shown in Figure 3A, by post-synthetic modification of the surface of Fe-MIL88B-NH_2_ with 1,4,7-triazacyclononane-1,4,7-triacetic acid (NOTA) and 5-amino-3-(pyrrolo [2,3-c]pyridin-1-yl)isoquinoline (defluorinated MK6240, DMK6240) and encapsulation of the tau protein aggregation inhibitor-methylene blue (MB), a drug system namely Fe-MIL-88B-NH_2_-NOTA-DMK6240/MB was prepared successfully. The cytotoxicity of this drug system was evaluated in SH-SY5Y cells using the MTT method. The result demonstrates that almost 83% of SH-SY5Y cells were still alive 24 h of incubation with 100 μg/ml of Fe-MIL-88B-NH_2_-NOTA-DMK6240 (Figure 3B). The 80% cell survival rate illustrates that Fe-MIL88B-NH_2_-NOTA-DMK6240/MB can effectively protect neurons from death (Figure 3C).

In addition to improve the loading efficiency of NMOFs by multiple coordination between the active compound and metal sites, physisorption by soaking the as-synthesized NMOFs nanocarriers into drug-containing solutions is another method to improve load efficiency of NMOFs. For example, Zou groups combined the synthesis of ZIFs and the encapsulation of target organic molecules in one pot (Zheng et al., 2016). The anticancer drug doxorubicin (DOX) guest molecules which adsorbed into the frameworks of ZIF-8 nanoparticles could be targeted delivery efficient without premature release. Besides doxorubicin, an autophagy inhibitor 3-methyladenine (3-MA) also could be capped in ZIF-8 via one-pot synthesis (Chen W H et al., 2018). The experiment showed that a remarkable capacity of 19.798 wt% can be achieved for 3-MA with ZIF-8 nanoparticles.

Larger molecule delivery encounters more challenges because of their size, surface charge, and component effects including peptides and proteins (Kato et al., 2019). The protein loading efficiency could be significantly improved by immersing NMOFs with a bigger channel size into a protein-containing solution to do physical adsorption (Lian et al., 2017). Farha groups immobilized insulin in NU-1000 due to the larger one-dimensional pores of NU-1000 (mesopores with size ∼30 Å and micropores with size∼12 Å in diameters), and acquired a high loading of ∼40 wt% in only 30 min (Chen X et al., 2018). The surplus insulin can be digested by pepsin and denatured by stomach acid. Conversely, when using NU-1000 as a nanocarrier for oral delivery of insulin, insulin@NU-1000 not only can keep the wholeness of insulin in gastric acid but also can prevent pepsin from entering the insulin to limit proteolysis simultaneously. Subsequently, they expanded the pore apertures of a series of Zr-based MOFs (termed NU-1000x, x = 3, 4, 5, 6, 7) from 3.3 to 6.7 nm. The expanded NU-1000x MOF structures were used to incorporate the lactate dehydrogenase (LDH) and then demonstrated the use of the captured protein in a cell-free biosynthetic catalytic system (Li B et al., 2018).

**FIGURE 4:**
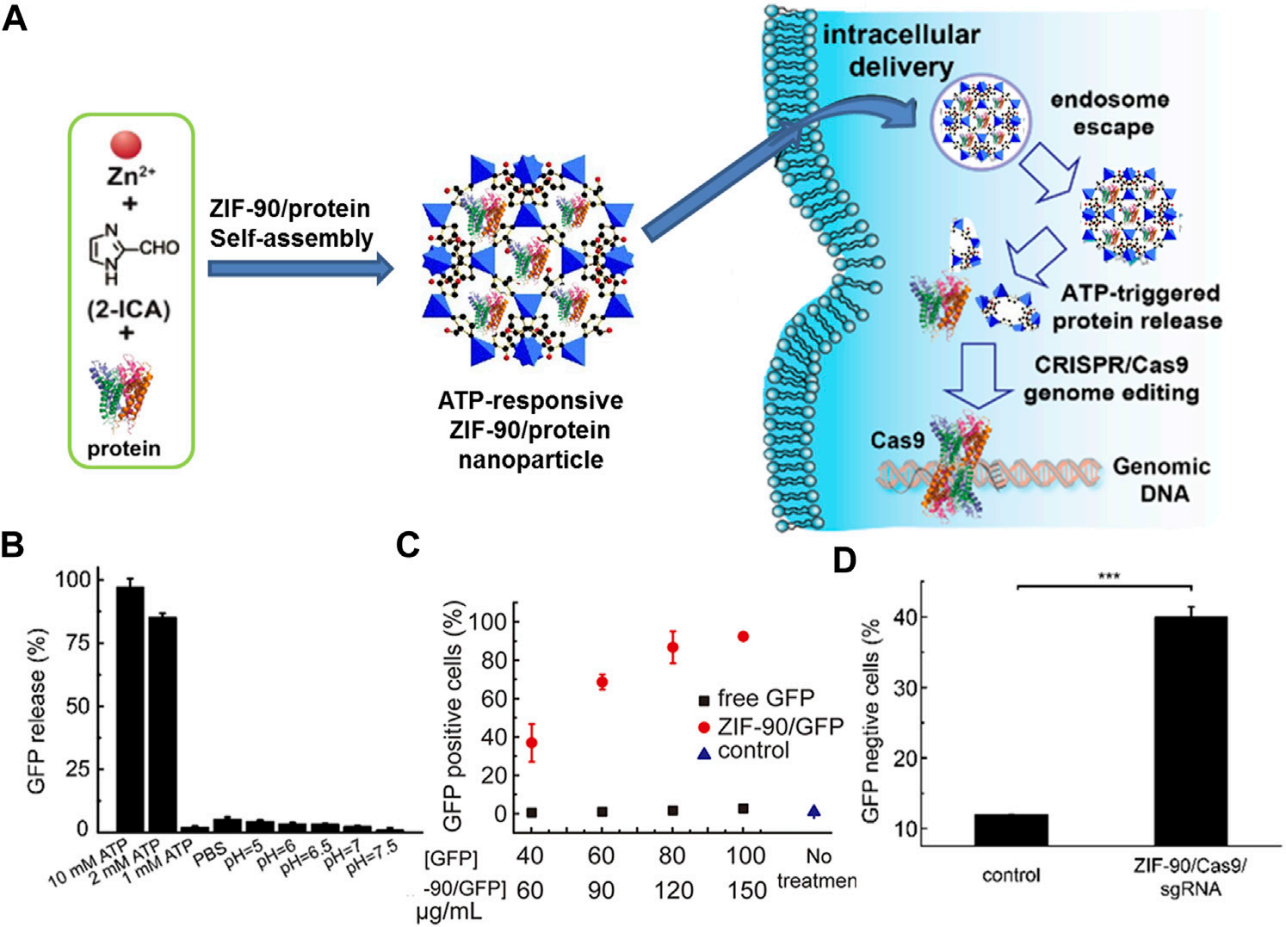
**(A)** The schematic illustration of the self-assembly of ZIF-90/protein and ATP-triggered protein release from ZIF-90 inside cells. **(B)** Selectivity of ATP triggered protein release from ZIF-90/GFP. **(C)** Cellular uptake efficiency of ZIF-90/GFP by HeLa cells. **(D)** The delivery of ZIF-90/Cas9 efficiently inhibits GFP expression of HeLa-GFP cells. HeLa-GFP cells were treated with ZIF-90/Cas9 nanoparticles (120 nM Cas9) alone with 60 nM sgRNA. Source: (Yang B et al., 2019).

Stimulus-responsive release: Although the terms of lower toxic, higher loading ability, and available organism permeability enable NMOFs as outstanding drug carriers, but the issues of premature drug release of NMOFs as nanocarrier is also extremely significant. Typically, stimuli-responsive factors of NMOFs are pH, biomolecules, temperature, and so on. Furthermore, loaded drugs of NMOFs could be activated by diverse stimuli (Lu K et al., 2016). For instance, acidic property of tumor tissue enables pH is one of the extensively studied stimuli for drug release. Willner and co-workers used ZIF-8 NMOF loaded glucose oxidase (GOx) and insulin, and the GOx-mediated aerobic oxidation of glucose yield H_2_O_2_ and gluconic acid (Chen Y et al., 2018). The high intracellular concentration of biomolecules in cell also can control drug release such as glutathione (GSH), adenosine triphosphate (ATP), and enzyme. Among numerous factors controlling drug release, disulfide bonds with GSH response is usually considered. Higher GSH level promote the reducing of the disulfide bond and finally set the drugs free. As an extremely important compound for organism, ATP usually used to offer energy and then promote biological processes deeper. Mao and workers reported the first case of using nanoscale ZIF-90 to specific subcellular mitochondria and image dynamics of mitochondrial ATP in cells (Deng et al., 2017). After embedding Rhodamine B (RhB) in ZIF-90, RhB/ZIF-90 nanoprobe with fluorescence is able to monitor the ATP level fluctuation in a various scope of cellular processes. Later, ZIF-90 with ATP-responsive platform was developed by the same group to deliver cytosolic protein and clustered regularly interspaced short palindromic repeats-associated protein 9 (CRISPR/Cas9) genome editing, as shown in Figure 4A (Yang X et al., 2019). An interesting finder is a slower green fluorescent protein (GFP) release was observed when treated the ZIF-90/GFP in 10 mM phosphate-buffered saline, which is differ to ZIF-8-based protein release with pH-responsive (Figure 4B). Intracellular delivery studies indicated that the ZIF-90/protein nanocarriers can deliver abundant of proteins into the cytosol, regardless of protein size and molecular weight (Figure 4C). Because of the competitive coordination between ATP and Zn^2+^, the ZIF-90/protein nanocarriers decompose in the existence of ATP and release protein. Such as, compared with non-treated HeLa cells, ZIF-90/protein treatment efficiently inhibited GFP expression in HeLa-GFP cells up to 40%, indicating an effective CRISPR/Cas9 genome editing upon ZIF-90/protein delivery (Figure 4D).

In general, release efficiency of drug molecules in temperature-responsive nanocarriers is more susceptible to temperature changes in living organisms. Sada groups reported an example of polymer-modified MOF tethering thermoresponsive polymer poly (N-isopropylacrylamide) (PNIPAM), showing thermally ON-OFF controllable behavior (Nagata et al., 2015; Chen et al., 2019). Two Zinc-based MOFs with formula Zn_16_ (ad)_8_ (TP)_8_O_2_(H_2_O)_8_·4HTP·36DMF·16H_2_O (DMF = N,N-Dimethylformamide) were designed by Qian groups (ad = adenine, H_2_TP = [1,1′:4′,1″-terphenyl]-4,4″-dicarboxylic acid for ZJU-64, and 2′,5′-dimethyl-[1,1′:4′,1″-terphenyl]-4,4″-dicarboxylic acid for ZJU-64-CH_3_) for loading anticancer drug methotrexate (MTX) (Lin et al., 2016). By an easy impregnation method, anticancer drug MTX could be incorporated into NMOFs and loading capacity of ZJU-64 is 13.45 wt%, of ZJU-64-CH_3_ is 10.63 wt%. Furthermore, equal amounts of MTX drugs released from MTX-loaded ZJU-64 and ZJU-64-CH_3_ at 37°C took 72 h at 60 °C only need about 1.5 and 6 h, proving a lurking application of ZJU-64 and ZJU-64-CH_3_ as temperature sensitive drug nanocarriers.

Sometimes a single factor is not enough to stimulate the drug release efficiency of NMOFs due to the complexity of organism. Multiple stimuli-responsive MOFs can be served as a better choice to increase drug release efficiencies. Yang and co-workers constructed the first example of mono-disperse mechanized NMOFs by post modification mechanism using stimuli responsive molecules in which pillarene-based supramolecular switches act as gatekeepers (Tan et al., 2015). This nanocarrier has exhibited pH- and/or competitive binding-triggered responded drug release. Deeper research on NMOFs-based nanocarriers with multipoint stimulus response have also been did by the same group in which carboxylatopillar [5]arene (CP5) cyclic structure act as locker. Electrostatic interaction between quaternary ammonium salt with positive charge that was coated on UiO-66-NH_2_ and the negatively charged CP5 cycles could produce [2]pseudorotaxanes as multipoint stimulus response to control drug release.

With the extensive research of NMOFs in the field of nanomedicine, some other factors that affect drug release also have been reported (Oh et al., 2015). Such as ion-responsive MOFs Zn_8_ (ad)_4_(BPDC)_6_O·2Me_2_NH_2_·8DMF·11H_2_O (bioMOF-1) (BPDC^2-^ = 4,4′-biphenyldicarboxylic acid) which was fabricated by Rosi group (An et al., 2009). Such anionic bio-MOF-1 could release a cationic drug (procainamide HCl) by the electrical reaction between cations and anions. Another case was given by Lin and co-workers, MIL-101 tethered drugs coated with silica shell could improve the drug release efficiency by prodrug hydrolysis because of the degradability of the silica shell in organism and phosphate buffered saline (PBS) solution (Taylor-Pashow et al., 2009). Moreover, pressure has been adopted for dominating drug release. Latterly, Qian groups designed a Zr-based MOF with high model drug diclofenac sodium (DS) loading amount up to 58.80 wt% (Jiang et al., 2016). The DS release kinetics could be controlled by regulating the pressure, resulting in a continual release between 2-8 days.

### Nanoscale Metal–Organic Frameworks as Phototherapeutic Agents for Cancer Therapy

*Cancer* is one of the major diseases detrimental to health and is a major public health problem worldwide. With the enormous progress made in cancer biology in the past few decades, a large number of anticancer therapeutics have been brought to the clinic including small molecule inhibitors, antibodies, chemotherapeutics, and nucleic acid drugs. However, owing to poor therapeutic effects, high risk of recurrence, intolerable side effects, and other reasons, conventional treatments often fail to meet clinical needs. Recently, phototherapeutic agents are widely used to treat cancer. However, due to the poor tissue penetration of light, pure phototherapy can only be used for superficial treatment. Adjustable structure, functional capabilities, biocompatibility, and biodegradability of NMOFs have made them a promising platform for cancer therapy. Noteworthy is the use of X-ray scintillating MOFs that constructed from metal cluster nodes of high atomic numbers (Z = 72 for Hf and Z = 40 for Zr). The Hf(IV) and Zr(IV) cations in the SBU act as antennas by absorbing X-ray photons and converting them to fast electrons through the photoelectric effect. The generated electrons scintillate/excite multiple anthracene-based optical emitters in the MOF through inelastic scattering, leading to efficient generation of detectable photons in the visible spectrum. Most importantly, NMOFs have the ability to preferentially deposit in tumors by enhancing permeability and retention (EPR) effect (Yang et al., 2020). In particular, the integration of photosensitizer to NMOFs not only addresses the limitations of photosensitizers, including aggregation, self-quenching, and uncontrollable *in vivo* but also improves loading efficiency, stability, and reduces cytotoxicity. Table 2 summarizes the form of cancer treatment of various NMOFs as phototherapeutic agents for cancer therapy.

**TABLE 2:** The form of cancer treatment of various NMOFs as phototherapeutic agents for cancer therapy.

NMOFs	Photosensitizers (PSs)	Light source	Therapeutic agents	Form of cancer treatment	Refs
PB@MIL-100(Fe)	Prussian blue (PB)	NIR		PTT	Wang B et al. (2016)
PB@mSiO_2_−PEG	Prussian blue (PB)	NIR	DOX	PTT	Su et al. (2016)
UiO-66@PAN	Polymer polyaniline (PAN)	NIR	-	PTT	Wang D et al. (2016)
PPy@MIL-100	Polypyrrole (PPy)	NIR	-	PTT and chemotherapy	Chen D et al. (2017)
MOF@HA@ICG	Indocyanine green (ICG)	NIR	-	PTT	Cai et al. (2017)
Hf_6_(*μ*_3_-O)_4_ (*μ*_3_-OH)_4_ (DBP)_6_	Porphyrin DBP	Visible light	^1^O_2_	PDT	Lu et al. (2014)
Hf_6_(*μ*_3_-O)_4_ (*μ*_3_-OH)_4_ (DBP)_6_	Porphyrin DBC	Visible light	^1^O_2_	PDT	Lu et al. (2015)
Hf-TCPP	Hf^4+^, Porphyrin	X-ray	^1^O_2_	PDT and RT	Liu et al. (2016)
PCN-224	Zr^4+^, Porphyrin	UV	^1^O_2_	PDT	Park et al. (2016a)
Zn-TCPP-BPDTE	Porphyrin TCPP	UV	^1^O_2_	PDT	Park et al. (2015)
UiO-66	TCPP	UV	^1^O_2_	PDT	Park et al. (2016b)
UiO-PDT	BODIPYs	Confocal laser	^1^O_2_	PDT	Wang et al. (2016a)
Hf-/Zr-based MOFs	[M_6_]-clusters (M = Hf or Zr)	X-ray, visible light	photons	PDT	Wang et al. (2014)
Hf-BPY-Ir/Ru	Hf atoms	X-ray	^1^O_2_	PDT	Lan et al. (2017)
IDOi@TBC-Hf	Hf atoms	LED	^1^O_2_ and IDOi	PDT and immunotherapy	Lu Y et al. (2016)
DBP-Hf	Hf atoms	LED	^1^O_2_ and IDOi	PDT and immunotherapy	Lu et al. (2018a)
g-C_3_N_4_@ZIF-8	g-C_3_N_4_	-	^1^O_2_ and IDOi	PDT and immunotherapy	Chen et al. (2015)
PCN-222	Porphyrin	-	^1^O_2_ and DOX	PDT and immunotherapy	Liu et al. (2017)
MIL-88B	Porphyrin	-	MB and DOX	PDT and chemotherapy	Sharma et al. (2017)
F127-MnO_2_-ZIF@DOX/C_3_N_4_	g-C_3_N_4_	NIR	^1^O_2_ and DOX	PDT and immunotherapy	Zhang Q et al. (2018)

Photothermal therapy (PTT): PTT is a popular noninvasive method for converting the incident light energy into heat for degrading tumors or killing cancer cell effectively via a light absorbing agent (Ni et al., 2020b). For NMOFs, three methods can be adopted for assembly multifunctional NMOF-based therapy for enhanced PTT. First, enhanced photothermal conversion efficiency by functionalizing the SBUs and bridging linkers of NMOFs. Second, incorporate the functional moieties into the framework of NMOFs via post-synthetic modification or self-assembly from pre-modified linkers for the combination therapies. Third, by encapsulated the multifunctional agents into NMOF frameworks, the treatment efficiency of PTT could be improved. For example, Prussian blue (PB) is a prototype of mixed-valence hexacyanoferrate with a formula of Fe^III^_4_ [Fe^II^(CN)_6_]_3_·nH_2_O (Fu et al., 2012). Biosafety and wider absorption range of PB NPs make it outstanding phototherapeutic agents that can be embedded in the structure of MOFs to formed nanocomposites for PPT. Chen and coworkers obtained a multifunctional PB@MIL-100(Fe) dual-MOFs nanomaterial for combined chemotherapy and PTT and steps for the synthesis of dual-MOFs therapeutic nanoparticle (Wang B et al., 2016). Subsequently, Zhang and coworkers acquired PB@mSiO_2_–PEG with a high antitumor drug DOX loading (Su et al., 2016). Under the irradiation of near-infrared laser, the constructured PB@mSiO_2_−PEG/DOX nanoplatform exhibits efficacy photothermochemical therapy for breast cancer.

**FIGURE 5:**
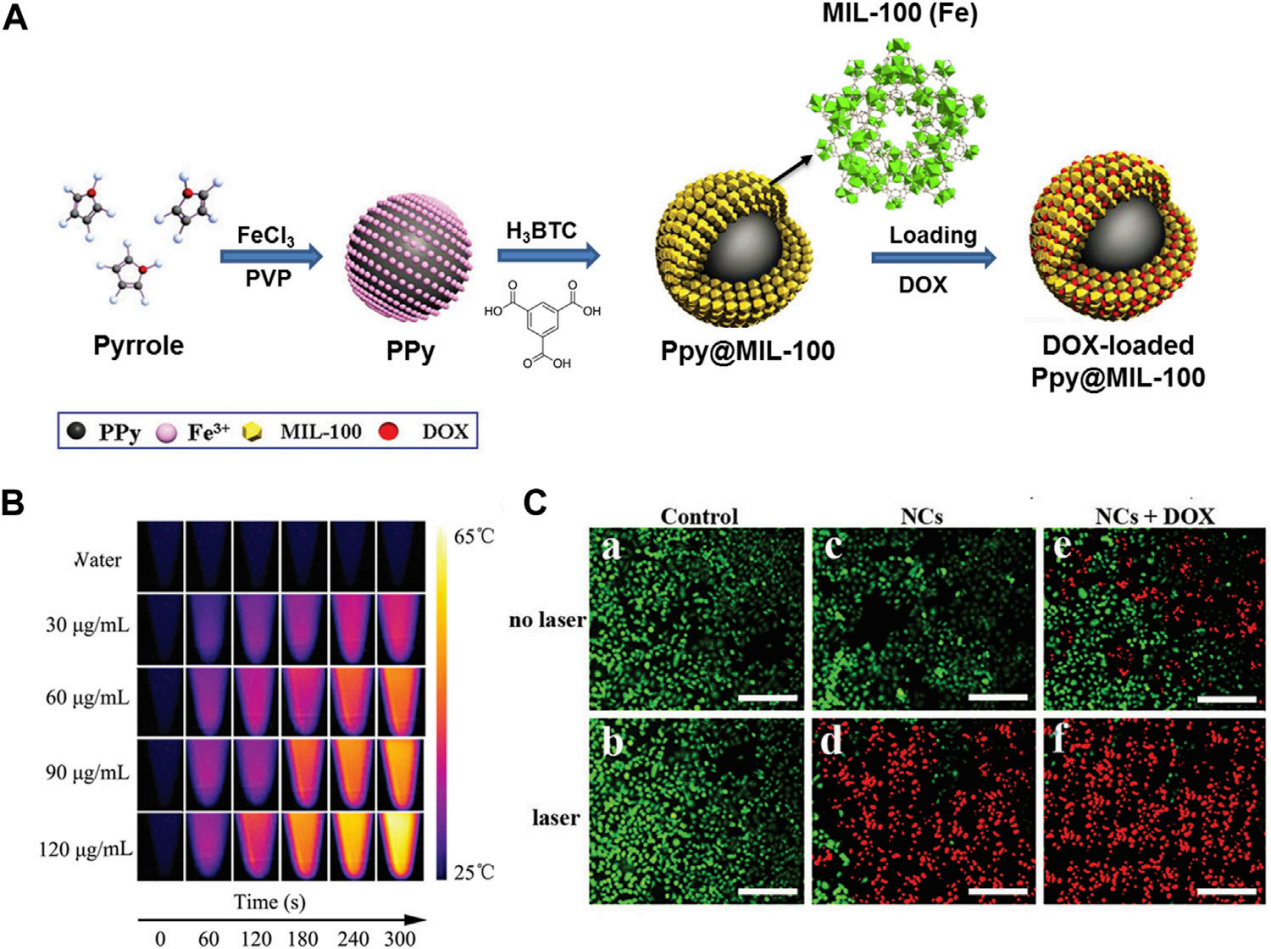
**(A)** The schematic illustration of synthesis process of PPy@MIL-100(Fe). **(B)** Photothermal images of PPy@MIL-100(Fe) solution at different concentrations exposed to the 808 nm laser (2 W cm^−2^) recorded at different time intervals (0, 1, 2, 3, 4 and 5 min). **(C)** Fluorescence images of HepG-2 cells with different treatments via staining with calcein AM/PI: a: control; b: laser irradiation only; c: PPy@MIL-100(Fe) NCs only; d: PPy@MIL-100(Fe) NCs with laser irradiation, e: DOX-loaded PPy@MIL-100(Fe) NCs only, f: DOX-loaded PPy@MIL-100(Fe) NCs with laser irradiation. Source: (Chen D et al., 2017).

Besides PB, NIR-absorbing polymer polyaniline (PAN) also exhibit strong absorption coefficients and excellent photostability that could be incorporated in the channels of NMOFs to construct nanocomposites for PTT upon NIR laser irradiation. Jing and co-workers synthesized a nanoscale polymer-MOF hybrid named UiO-66@PAN, in which PAN had the NIR-absorbing ability (Wang D et al., 2016). The UiO-66@PAN is effective for PTT based cancer treatment both *in vivo* and *in vitro*. As a result of higher photothermal transformation efficiency and outstanding biocompatibility, Polypyrrole (PPy) incorporated nanomaterial has been proved to be efficient for PTT. The fabrication process of PPy@MIL-100 was shown in Figure 5A, in which PPy and FeCl_3_·6H_2_O acted as a catalyst and PVP as stabilizer. The PPy core acts as a PTT agent while an organic PAI agent for deep tissue imaging. The MIL-100 shell was applied for loading DOX drugs. It was suggested that polypyrrole was formed by Fe(III) ions induced oxidative polymerization of PPy, then the combination of Fe(III) ions and polypyrrole surface promoted the formation of MIL-100 shell on PPy. Moreover, PPy@MIL-100 supported pH- and light controlled drug release, dual-modal imaging, and combined PTT and chemotherapy (Figures 5B,C). Besides this, Wang groups reported a dual-modal imaging core-shell nanomaterial PPy@MIL-100 for combined chemotherapy and PTT (Chen R et al., 2017). Indocyanine green (ICG) is the NIR optical dye and can be used for clinical applications. For example, Chen groups designed a multifunctional nanoplatform MOF@HA@ICG based on hyaluronic acid (HA) for imaging-guided PTT (Cai et al., 2017). Hovever, the lacked of sensitivity and specificity in cancer treatment limited its application.

**FIGURE 6:**
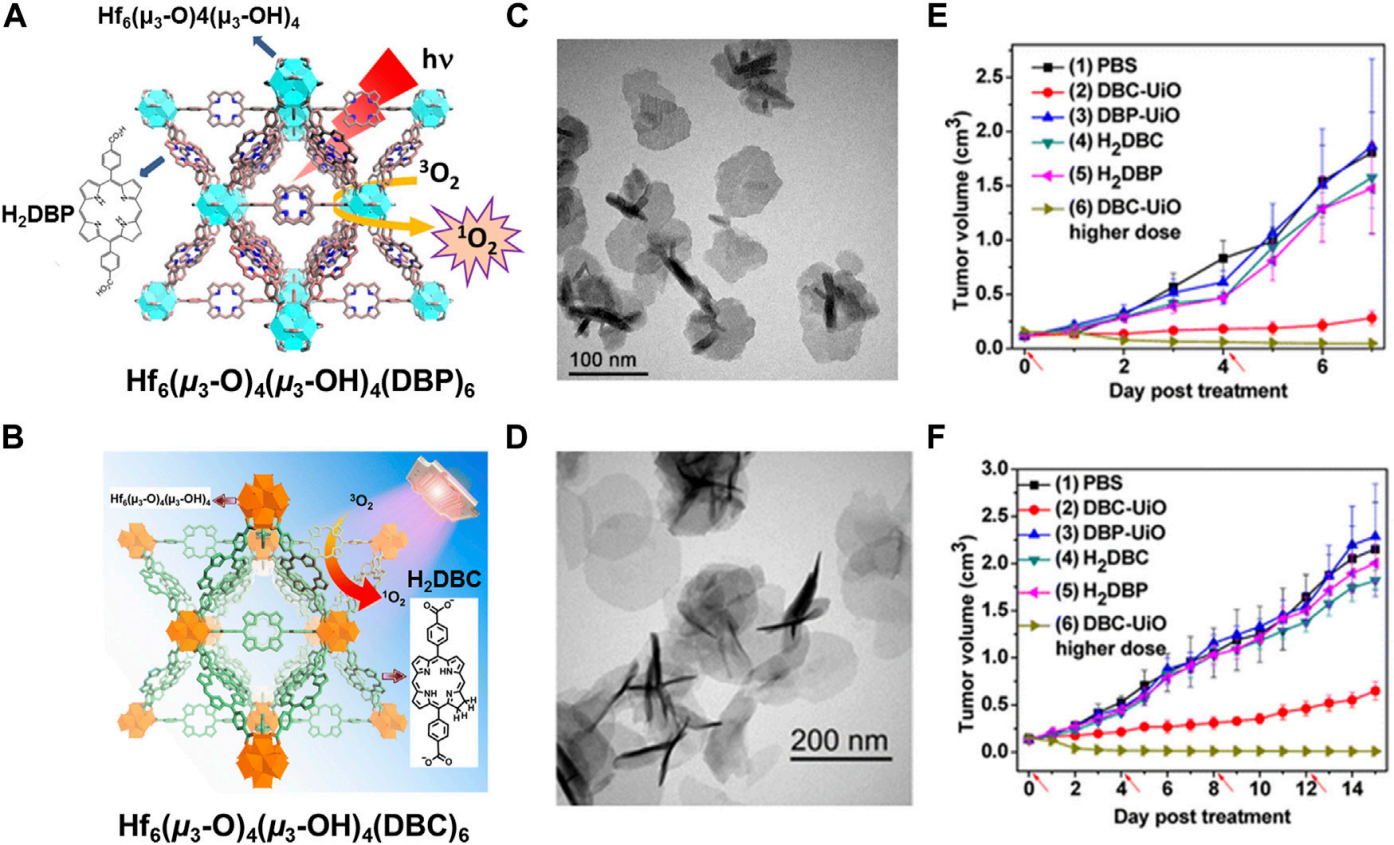
**(A)** The structure of Hf−DBP NMOF and the schematic description of ^1^O_2_ generation process. **(B)** The structure of Hf−DBC NMOF and the schematic description of ^1^O_2_ generation process. **(C)** TEM image of DBP−UiO with nanoplate morphology. **(D)** TEM image of DBC−UiO with nanoplate morphology. *In vivo* PDT efficacy on CT26 and HT29 tumor-bearing mice: **(E)** Tumor growth inhibition curve after PDT treatment by DBP−UiO. **(F)** Tumor growth inhibition curve after PDT treatment by DBC−UiO. Source: (Lu et al., 2014; Lu et al., 2015).

Photodynamic therapy (PDT): Photodynamic therapy (PDT) is more effective anticancer treatment that depends on nontoxic photosensitizer (PS) to yield reactive oxygen species (ROS) to inhibit cancer cells (Horne and Cronjé, 2017). The first way of constructing NMOFs for PPT is using the nontoxic photosensitizer as building blocks. In 2014, Lin and co-workers first literates a Hf-porphyrin NMOFs with formula of Hf_6_(*μ*_3_-O)_4_ (*μ*_3_-OH)_4_ (DBP)_6_ (H_2_DBP = 5,15-di (*p*-benzoato)-porphyrin) by a solvothermal strategy, as a significant photosensitizer for PDT (Lu et al., 2014). Thanks to position separation of porphyrin ligands, Hf_6_(*μ*_3_-O)_4_ (*μ*_3_-OH)_4_ (DBP)_6_ could efficiently generate ^1^O_2_ to enhance intersystem crossing by heavy Hf centers, and realize ^1^O_2_ invasive from the interior of MOFs to the cell through porous nanoplates, as shown in Figures 6A,C. Porous NMOF structure impelled Hf_6_(*μ*_3_-O)_4_ (*μ*_3_-OH)_4_ (DBP)_6_ remarkable PS loading up to 77 wt%. Moreover, in NMOF-treated group with 180 J cm^−2^ light dose, two quarters mouse had their tumors eradicated while the other two achieved 98% tumor regression, on contrary, the ligand control failed to inhibit the tumor growth at the same dose (Figure 6E). Then, the same group used another hematoporphyrin derivative 5,15-di (*p*-benzoato)-chlorin (H_2_DBC) as ligand to coordinate with Hf and finally afforded a UiO framework of Hf_6_(*μ*_3_-O)_4_ (*μ*_3_-OH)_4_ (DBC)_6_ by solvothermal reaction (Figure 6B) (Lu et al., 2015). The Hf_6_(*μ*_3_-O)_4_ (*μ*_3_-OH)_4_ (DBC)_6_ NMOF is isostructural to Hf_6_(*μ*_3_-O)_4_ (*μ*_3_-OH)_4_ (DBP)_6_, showing a thin nanoplate morphology with thicknesses of 3.3–7.5 nm (Figure 6D). Importantly, compared to Hf_6_(*μ*_3_-O)_4_ (*μ*_3_-OH)_4_ (DBP)_6_, Hf_6_(*μ*_3_-O)_4_ (*μ*_3_-OH)_4_ (DBC)_6_ exhibits a 13 nm red shift and an 11-fold increase in the extinction coefficient of the lowest-energy Q-band that providing improved light penetration through tissues. Furthermore, Hf_6_(*μ*_3_-O)_4_ (*μ*_3_-OH)_4_ (DBC)_6_ is 3 times as efficient as Hf_6_(*μ*_3_-O)_4_ (*μ*_3_-OH)_4_ (DBP)_6_ in generating ^1^O_2_ and exhibits much higher PDT cytotoxicity in two colon cancer cell lines (Figure 6F).

In addition to the above porphyrin derivatives, 5,10,15,20-tetrakis (4-carboxyphenyl) porphyrin (H_4_TCPP) is a commercially available photosensitizer applied in PDT (Bůžek et al., 2017). The symmetrical molecular structure of TCPP and the strong coordination ability of its four carboxyl groups to metal ions could facilitate the formation of MOF structure. Liu groups obtained a NMOF Hf-TCPP composed by Hf^4+^ and TCPP and proved the ability for efficient PDT by intravenous injection (Liu et al., 2016). Hf-TCPP displayed spherical morphology structure and TCPP in Hf-TCPP act a photosensitizer to allow PDT. In addition, strong X-ray attenuation ability of Hf^4+^ could act as a radio-sensitizer to enhance radiotherapy (RT). Zhou group reported a series of Zr^IV^-based porphyrinic NMOF (PCN-224) with different sizes (Park et al., 2015). The author indicated that modifing the surface of PCN-224 with folic acid could improve cell uptake of NMOFs and exhibited higher PDT efficacy.

Dithienylethene derivative, 1,2-bis(2-methyl-5-(pyridin-4-yl)thiophen-3-yl)cyclopent-1-ene (BPDTE) could serve as a photochromic switch to adopt reversible 6π electrocyclic reaction under photoirradiation at distinct wavelengths. The switch effect of BDPTE induced by light isomerization show special absorption characteristics, and can supply diverse energy transfer ways for photosensitizers to generate ^1^O_2_. Meanwhile, dipyridyl head in BPDTE make it possible for forming pillar-layer structure with TCPP to produce ^1^O_2_. Zhou groups constructed Zn(II) based MOF (Zn-TCPP-BPDTE) by solvothermal reaction of TCPP (no metal), BPDTE, and Zn(NO_3_)_2_·6H_2_O. In Zn-TCPP-BPDTE, paddlewheel-type Zn cluster linked TCPP via Zn-O bonds and linked BPDTE through the Zn–N bonds (Park et al., 2015). Upon UV irradiation, Zn-TCPP-BPDTE could present switch effect owing to the photochromic property of BPDTE. Moreover, the “on” state of Zn-TCPP-BPDTE without impacting the ^1^O_2_ generation of TCPP, but the BPDTE in “off” state of Zn-TCPP-BPDTE extinguished the S_1_ state of TCPP to decrease ^1^O_2_ generation. Later, they used the same technique obtained a UiO-66 NMOF-based ^1^O_2_ generation platform in which 1,2 bis(5-(4-carbonxyphenyl)-2-methylthien-3-yl)cyclopent-1-ene (BCDTE) as the photochromic switch and TCPP ligand as the PS (Park et al., 2016a). The cytotoxicity test showed that PDT cytotoxicity in the “on” state of BCDTE doped UiO-66 is ∼90%, while in the “off” state only 10%. This result proved the method of encapsulating photochromic molecules and PSs together into NMOFs to regulate ^1^O_2_ generation efficacy and finally impact *in vitro* PDT.

Ligand exchange offers another way for fabricating NMOFs for PDT. Xie and co-workers incorporated the carboxyl-modificated diiodo-substituted BODIPYs (I2-BDP) into UiO-66 to elaborate the PDT into the product of UiO-PDT through solvent-assisted ligand exchange (Wang et al., 2016a). Structural analysis shows that there was 12.5 mol% of benzenedicarboxylate linker was exchanged by carboxy-I2-BDP in UiO-PDT and affording a PS loading of 31.4 wt%. Both UiO-PDT and carboxy-I2-BDP had the ability of crossing the cell membrane to accumulate in the cytoplasm through Confocal laser scanning microscopy study. The penetration of light affects efficiency of NMOFs for PDT as well as the building blocks in anticancer treatment. Compared with visible light and near-infrared, X-rays have stronger penetrability in organism and have been widely employed in cancer treatment (Song et al., 2018). Lin and co-workers did outstanding work on efficiently converting X-ray to visible-light luminescence of NMOFs for PDT. In 2014, they first designed and synthesized X-ray Hf-/Zr-based MOFs with formula of M_6_ (*μ*_3_-O)_4_ (*μ*_3_-OH)_4_ (carboxylate)_12_ (M = Hf or Zr) (Wang et al., 2014). The high atomic number of [M_6_]-clusters in the SBUs acts as effective X-ray antenna by absorbing X-ray photons and transforming them into fast electrons. Once the [M_6_]-clusters assimilated X-rays in the energy range of 20–200 keV, the outer electrons of M^4+^ were emitted in the form of fast electrons to produce visible light. The X-ray-induced emission spectra exhibited that the emission peaks of M_6_ (*μ*_3_-O)_4_ (*μ*_3_-OH)_4_ (carboxylate)_12_ was in the visible range of 400–600 nm. Obviously, this visible light had potential for serve as a PDT light source. In 2017, they built two Hf-based nanomaterials Hf-BPY-Ir or Hf-BPY-Ru as powerful PSs for effective X-PDT of colon cancer models in which [Hf_6_O_4_(OH)_4_(HCO_2_)_6_] as SBUs and Ir [bpy (ppy)_2_]^+^- or [Ru (bpy)_3_]^2+^- as ligands (bpy = 2,2’-bipyridine, ppy = 2-phenylpyridine) (Lan et al., 2017). Hf-BPY-Ir was obtained by reacting [Ir (ppy)_2_Cl]_2_ and Hf-based MOF in a post-synthesis modification strategy (H_3_BPY = 4′,6′-dibenzoato-[2,2′-bipyridine]-4-carboxylic acid). Similarly, Hf-BPY-Ru nanomaterial were designed using Ru (bpy)_2_Cl_2_. Heavy Hf atoms in the SBUs absorb X-rays efficiently and transfer the energy to Ir [bpy (ppy)_2_]^+^ or [Ru (bpy)_3_]^2+^ units to lead to PDT by generating ROS. The fabricated cell experiment showed that these two nanomaterials had an excellent inhibitory effect on CT26 and MC38 cells *in vitro*, while *in vivo* experiments proved the efficient penetration of X-rays into tumor to enable efficient deep PDT which was performed in amice.

Combined theranostic strategies: Although PTT and PDT can strengthen by diverse strategies; the effect of monotherapy is still not satisfactory. Up to now, cancer treatment has gradually shifted from monotherapy to combination therapy which means integrating of two or more treatments for synergistic effects (Zhang et al., 2020). In 2016, Lin and co-workers obtained IDOi@TBC-Hf nanomaterial with chlorin derivative, 5,10,15,20-tetra (pbenzoato) chlorin (H_4_TBC) and Hf_6_(*μ*_3_-O)_4_ (*μ*_3_-OH)_4_ in which IDOi was loaded (Lu Y et al., 2016). Through the combination of NMOF-enabled PDT and IDOi-based immunotherapy, IDOi@TBC-Hf platform makes a possible cancer therapy strategy. Mechanistic studies suggested that ROS were generated upon irradiation after the local injection of IDOi@TBC-Hf leading immunogenic cell death and the release of tumor associated antigen. Subsequently, the same group obtained a series of IDOi-loaded NMOFs as a treatment therapy that synergized checkpoint blockade immunotherapy and low doses of X-ray-excited PDT to acquire effective local control and abscopal effects in mice models, including DBP-Hf (H_2_DBP = 5,15-di (*p*-benzoato)porphyrin), TBP-Hf (H_4_TBP = 5,10,15,20-tetra (*p*-benzoato)porphyrin), DBA-Hf_6_ and DBA-Hf_12_ (H_2_DBA = 2,5-di (*p*-benzoato)aniline) (Lu et al., 2018b). The combination of X-ray-induced PDT and immunotherapy can not only reduce the possible various effects of local X-ray radiation but also eradicate both treated primary tumors and untreated distant tumors efficiently via systemic antitumor immune responses.

**FIGURE 7:**
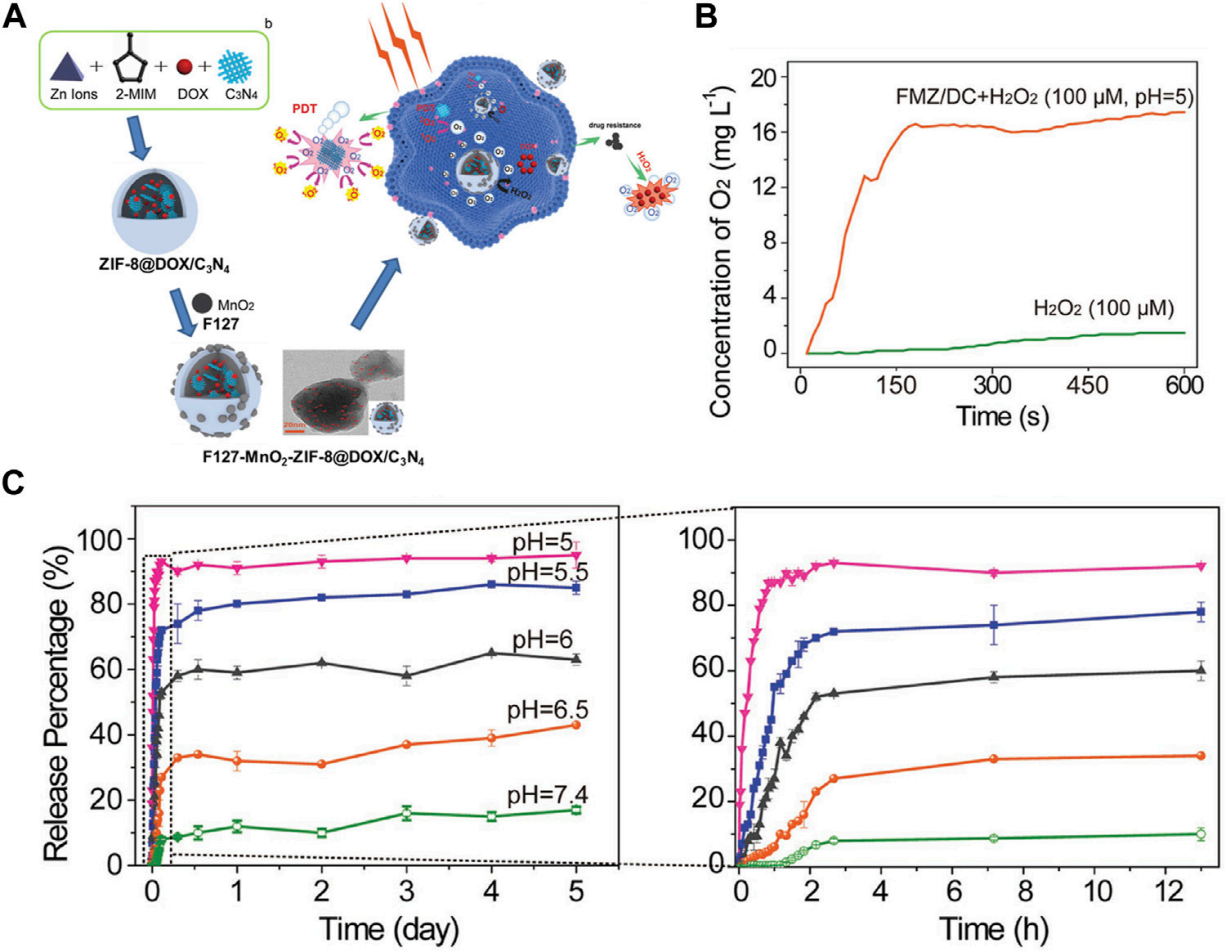
**(A)** The schematic illustration of the fabrication of F127-MnO_2_-ZIF@DOX/C_3_N_4_ and F127-MnO_2_-ZIF@DOX/C_3_N_4_ with ^1^O_2_ generation enhancing the chemo-photodynamic therapy. **(B)** *In vitro* evaluation of ^1^O_2_ generation of FMZ/DC in H_2_O_2_ solution (10–4 m) under pH = 5. **(C)** The pH-responsive release of DOX from FMZ/DC determined by UV–vis spectrophotometry. Source: (Xu et al., 2017).

The major defects of chemotherapy are intolerable side effects, systemic toxicity, and multidrug resistance. When united the PDT and chemotherapy, PDT-induced photochemical internalization can significantly improve the efficacy of chemotherapy (Ni et al., 2018). Such as the encapsulation of anticancer drug DOX into the NMOF with photodynamic effect not only could meet high loading rate of DOX but also could facilitate the induction of ^1^O_2_ production because of the high content of PS in the NMOFs. Lee and co-workers produced a core-shell structure g-C_3_N_4_@ZIF-8 for DOX drug delivery (Chen et al., 2015). The anticancer efficacy of g-C_3_N_4_@ZIF-8 enhanced significantly by combination DOX with g-C_3_N_4_. Besides ZIF-8, PCN-222 was also used as a nanocarrier for loading DOX and PDT (Liu et al., 2017). 109 wt% loading rate of DOX was obtained due to the larger pore channel (1.75V 1.75 nm). In addition, ^1^O_2_ production could be facilitated by the high concentrations of porphyrin (59.8 wt%) in the PCN-222. Similarly, Roy and co-workers acquired a combination of PDT and chemotherapy by incorporated MB and DOX into the micropores of MIL-88B (Sharma et al., 2017). In order to effectively strengthen the antitumor effect, Wang and co-workers obtained F127-MnO_2_-ZIF@DOX/C_3_N_4_ nanomaterial with integration of chemotherapy and aerobic PDT, which had a fairly good curative effect on hypoxic tumors (Zhang Q et al., 2018). Particularly, the g-C3N4 PS and the DOX drugs were incorporated into the ZIF-8 by electrostatic interactions finally obtained F127-MnO_2_-ZIF@DOX/C_3_N_4_ nanomaterial (Figure 7A). F127 improved the biocompatibility the nanomaterial, promoting its accumulation in cancer (Figure 7B). ZIF-8 offered pH-responsive characteristics for the nanomaterial: releasing DOX under acidic conditions, reducing side effects, and reducing nonspecific drug release (Figure 7C). In addition, the loaded MnO_2_ nanodots could continuously catalyze the decomposition of endogenous H_2_O_2_ to produce O_2_, and greatly enhance the PDT effect.

### Nanoscale Metal–Organic Frameworks as Fluorescent Probe for Biomedical Sensing and Imaging

Biomedical sensing and imaging have been proved as prospecting pattern for detection multiple complexes selectively and rapidly as well as environmental safety (Dong et al., 2019). Luminescent characteristics together with size/shape selective sorption properties of NMOFs make them potential fluorescent probe for biomedical sensing and imaging under different mechanisms. So far, various NMOFs were explored for detecting DNA/RNA, enzyme activity and small-biomolecules, as well as magnetic resonance imaging (MRI), X-Ray computed tomography (CT), positron emission tomography (PET) and optical imaging, which are useful techniques for clinical diagnosis.

Biomedical sensing: Through overcoming the self-quenching and leaching problems of many small molecular biosensors, NMOF-based sensors realize high sensitivity, high precision, and high resolution in subcellular bioprobes. Recently, a lot of studies have reported to describe the NMOFs as sensitive and selective sensors for screening biological macromolecules like nucleic acids (Yang Y et al., 2017), and proteins (Xu et al., 2017), small biomolecules including glucose (Chen et al., 2016), amino acids (Wang et al., 2015), and so on in cellular and subcellular sensing, as shown in Table 3.

**TABLE 3:** Various NMOFs as sensitive and selective sensors for screening biological macromolecules.

NMOFs	Probe units	Biomolecules	Detection limit	Detection mechanism	Refs
[Cu(H_2_dtoa)]	FAM-labeled probe	HIV-1 (DNA), Thrombin	-	PET	Zhu et al. (2013)
[Cu_3_(Cmdcp)_2_ (dps)_4_·(H_2_O)_4_(SO_4_)]_n_	FAM-labeled probe	HIV-1 (dsDNA) and Sudan virus RNA	196 pM for DNA, 73 pM for RNA	PET	Yang et al. (2015)
{[Dy(Cmdcp)(H_2_O)_3_](NO_3_)·2H_2_O}_n_	-	Ebolavirus RNA	160 pM for RNA	π-stacking and/or hydrogen bonding	Qin et al. (2016)
{[La_4_(Cmdcp)_6_(H_2_O)_9_]}_n_	FAM-labeled probe	P-DNA	-	PET	
{[La_2_(Cbdcp)_3_(H_2_O)_10_]}_*n*_	FAM-labeled probe	DNA or RNA	-	PET	Yang S P et al., 2017, Zhao et al. (2016a), Zhao et al. (2016b)
{[Zn(HCbdcp)_2_]H_2_O}_n_					
[Cu(dcbb)_2_]_n_					
{[Cu(dcbb)_2_(H_2_O)_2_]·10H_2_O}_n_					
UiO-66-NH_2_	FAM-labeled probe	HIV-1 (DNA)	-	Electrostatic and π-π stacking	Fang et al. (2014), Mejia-Ariza et al. (2017), Zhao H X et al. (2016), Ling et al. (2015)
UiO-66					
MIL-101					
MIL-88A					
[Gd (TIA)(HCOO)]_n_					
{[La_2_ (TDA)_3_]·2H_2_O}_n_					
Cd(L)·(HDMA)_2_ (DMF)(H_2_O)_3_					
Zn(L)·(HDMA)_2_ (DMF)(H_2_O)_6_					
[Cd (ATC)(H_2_O)_2_]_n_	Functional (-COOH/-NH_2_) groups	anti-BSA	7 mg/ml	-	Kumar et al. (2014)
[Cu(mal)(bpy)]·2H_2_O	FAM-labeled probe	glycine (Gly) and serine (Ser)	0.81 μg ml^−1^ for Gly, 1.51 μg ml^−1^ for Ser	-	Li S Y et al. (2017)
{[Cd(L)(bpy)]·DMA·5H_2_O}_n_	π-conjugated naphthyl moiety	penicillamine (PEN)	23.72 μM^−1^ for D-PEN, 10.13 μM^−1^ for L-PEN	-	Zhang W et al. (2018)
R-UiO	Oxygen-responsive phosphorescent ligand	ROS	-	-	Xu et al. (2016)
Zr_6_O_4_(OH)_8_(H_2_O)_4_(CTTA)_8/3_	-	NZF and TNP Antibiotics	58 ppb NZF, 23 ppb TNP	Electron transfer and energy transfer	Wang et al. (2016b)
Zr_6_O_4_(OH)_8_(H_2_O)_4_ (TTNA)_8/3_			58 ppb NZF, 23 ppb TNP		
[Cd_2_Na(L)(BDC)_2.5_]·9H_2_O	-	NZF antibiotics	162 ppb	Electron transfer and energy transfer	Zhao et al. (2017)
[Cd_2_(L)(2,6-NDC)_2_]·DMF·5H_2_O			75 ppb		
[Cd_2_(L)(BPDC)_2_]·DMF·9H_2_O			60 ppb		

The most exciting stories of the biomolecular sensing applications of NMOFs have been found from screening nucleic acid molecules, such as DNAs and RNAs. The detecting of DNAs and RNAs by NMOFs were mainly realized by anchoring NMOFs to nucleic acid molecules detectors. For instance, in order to detect the human immunodeficiency virus (HIV-1), a N,N’-bis(2-hydroxyethyl) dithiooxamidato containing NMOF named [Cu(H_2_dtoa)] has been synthetized (Zhu et al., 2013). The sensing mechanism of this detecting platform is to regenerate the photoluminescence intensity of the carboxyfluorescein (FAM)-labeled probe DNA by binding specific DNA with MOFs. With the similar quenching principle, NMOF with formula of [Cu_3_(Cmdcp)_2_ (dps)_4_·(H_2_O)_4_(SO_4_)]_n_ (H_3_CmdcpBr = N-carboxymethyl-3,5-dicarboxylpyridinium bromide; dps = 4,4′-dipyridyl sulphide) was also used for detecting of HIV-1 double-stranded DNA (dsDNA) and Sudan virus RNA (Yang et al., 2015). Later, the research group used lanthanides ions and the identical inorganic linker to obtained {[La_4_(Cmdcp)_6_(H_2_O)_9_]}_n_ and {[Dy(Cmdcp)(H_2_O)_3_](NO_3_)·2H_2_O}_n_ with water stability (Qin et al., 2016). The coordinated water molecules and free NO_3_^-^ anions on the channels of {[Dy(Cmdcp)(H_2_O)_3_](NO_3_)·2H_2_O}_n_ can induce electrostatic and hydrogen bonding interactions with FAM-tagged probe DNA. Moreover, the transient complex formed by NMOFs and FAM-tagged probe DNA served as high efficiency and selectivity fluorescent probes for sensing Ebolavirus RNA. MOF {[La_4_(Cmdcp)_6_(H_2_O)_9_]}_*n*_ can absorb FAM-tagged probe DNA and quench the fluorescence of FAM through a photo-induced electron transfer (PET) process. Similarly, other water-stable NMOFs {[La_2_(Cbdcp)_3_(H_2_O)_10_]}_*n*_ (H_3_CbdcpBr = *N*-(4-carboxybenzyl)-3,5-dicarboxylpyridinium bromide), {[Zn(HCbdcp)_2_]H_2_O}_n_, [Cu(dcbb)_2_]_n_ (H_2_dcbbBr = 1-(3,5-dicarboxybenzyl)-4,4′-bipyridinium bromide) and {[Cu(dcbb)_2_(H_2_O)_2_]·10H_2_O}_n_ also have been reported for sensing virus DNA or RNA by absorbing FAM-tagged probe DNA and inhibited the fluorescence of FAM by PET (Zhao et al., 2016a; Zhao et al., 2016b; Yang S P et al., 2017).

Additionally, UiO-66-NH_2_, UiO-66, MIL-101, MIL-88A, {[La_2_ (TDA)_3_]·2H_2_O}_n_ (TDA = 2,2′-thiodiacetic acid), [Gd (TIA)(HCOO)]_n_ (TIA = 5-triazole isopthalate), Cd(L)·(HDMA)_2_ (DMF)(H_2_O)_3_ and Zn(L)·(HDMA)_2_ (DMF)(H_2_O)_6_) (H_4_L = bis-(3,5-dicarboxy- phenyl)terephthalamide, DMA = N,N-dimethylacetamide) (Fang et al., 2014; Ling et al., 2015; Zhao H X et al., 2016; Mejia-Ariza et al., 2017) have also been used as fluorescent probes for DNA/RNA and is able to distinguish complementary and mismatched target sequences with good selectivity and sensitivity. Besides biological molecular DNA/RNA, proteins can also be identified by NMOFs. MOF [Cd (ATC)(H_2_O)_2_]_n_ (ATC = 2-aminoterephthalic acid) has recently been proposed as a molecular sensing platform for the fluorescence quenching based detection of bovine serum albumin (anti-BSA) through activate -COOH/-NH_2_ groups followed by the attachment of proteins (Kumar et al., 2014). Limit of detection or the proposed detection is found to be 7 mg/ml that is comparable to the quantum dot based detections.

Besides the DNA/RNA and proteins biological macromolecular, NMOFs can also serve as fluorescence sensors to distinguish amino acids (Wang and Wei, 2016). Pioneering work on amino acids recognition includes the detection of dipicolinic acid (DPA) molecules, a structural constituent of *bacillus* anthrasis spores and many other virulent bacteria. Particularly, MOF named [Cu(mal)(bpy)]·2H_2_O (mal = D, l-malic acid) was constructed by Kong groups, a fluorescence probe experiment indicated that this MOF could act as fluorescence sensor to recognize glycine (Gly) and serine (Ser) and with low detection limits 0.81 μg ml^−1^ for Gly and 1.51 μg ml^−1^ for Ser (Li Y et al., 2017). The sensing principle is that fluorophore-labeled ssDNA can be adsorbed in [Cu(mal)(bpy)]·2H_2_O, and then quenching of the fluorescence. In term of chiral precursors 4,4’-((naphthalene-1,4-dicarbonyl)bis (azanediyl))dibenzoic acid (H_2_L) and bpy, a 3D homochiral LMOF {[Cd(L)(bpy)]·DMA·5H_2_O}_n_ was successfully obtained by Zhang and co-works and can be used as detecting platform for distinguishing _D_/_l_-penicillamine (PEN) by the π-conjugated naphthyl groups as fluorescent emission center (Zhang W et al., 2018). Once excited {[Cd(L)(bpy)]·DMA·5H_2_O}_n_ under 340 nm, a quite strong fluorescence was obtained. By adding the D-PEN or L-PEN into this platform, the luminescence intensity quenched obviously. The linear range of this fluorescence sensor is from 20 to 167 μM, and the detection limits of D-Pen and L-Pen are 23.72 μM^−1^ and 10.13 μM^−1^, respectively.

**FIGURE 8:**
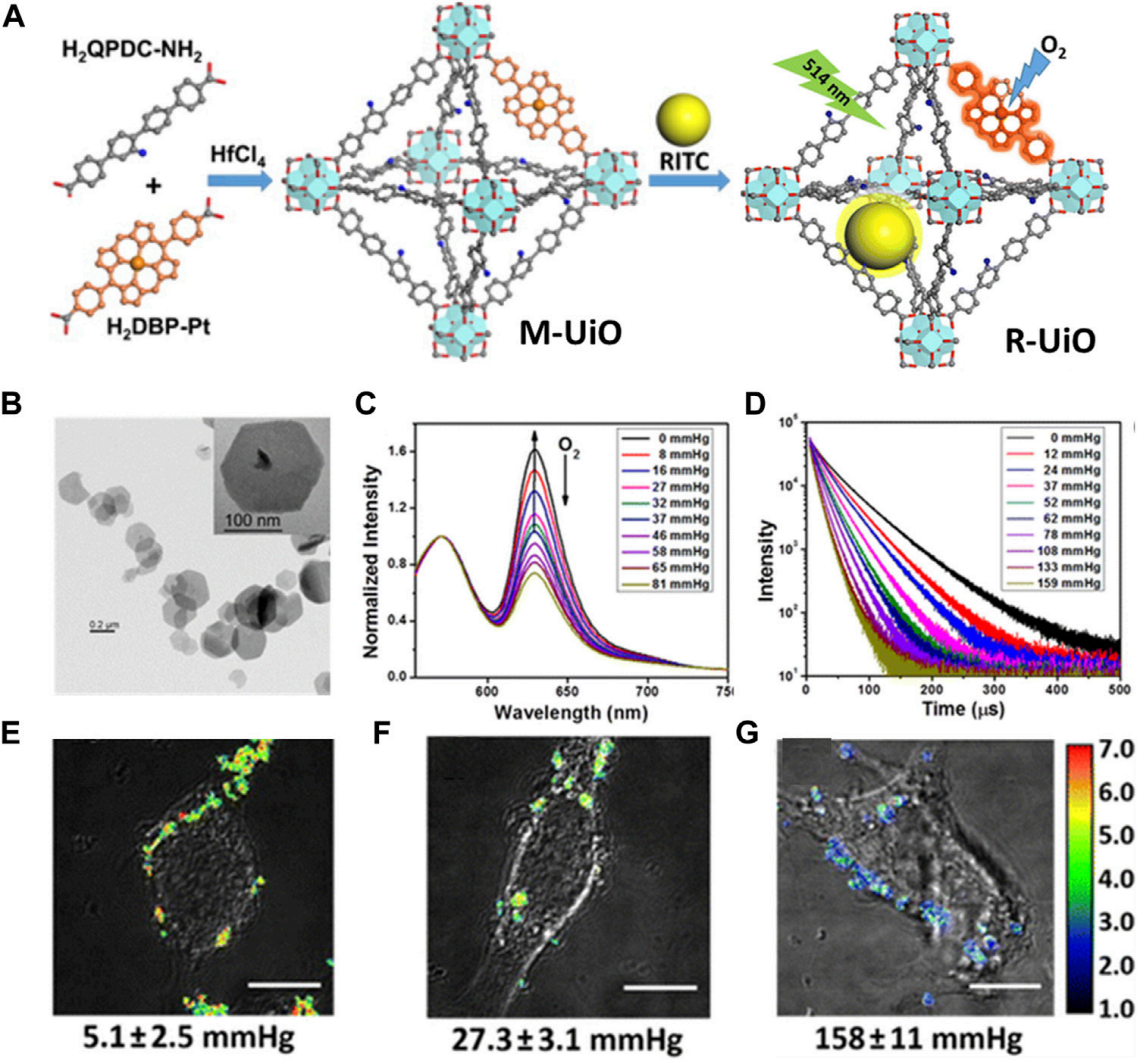
**(A)** The schematic illustration of synthesis process of mixed-ligand M-UiO NMOF and R-UiO NMOF; Schematic illustration of R-UiO NMOF offers oxygen generation under 514 nm light irradiation. **(B)** TEM images of R-UiO. **(C)** Emission spectra (λex = 514 nm) and **(D)** phosphorescent decays (λex = 405 nm) of R-UiO-1 in HBSS buffer under various oxygen partial pressures. Ratiometric luminescence imaging (λex = 514 nm) of CT26 cells after incubation with R-UiO-2 under **(E)** hypoxia, **(G)** normoxia, and **(F)** aerated conditions. Scale bar: 10 μm. Source: (Xu et al., 2016).

A variety of diseases cause hypoxia. PDT, radiation therapy and other common cancer treatment are all heavily oxygen-dependent treatments, so intracellular ROS sensing is greatly important and necessary. A phosphorescence/fluorescence dual-emissive NMOF was designed by Lin groups, namely, R-UiO, which could be used as an intracellular ROS detector (Xu et al., 2016). The R-UiO was stable, porous, crystalline, and dual emissive (Figures 8A,B). The mix-ligand R-UiO including an oxygen-responsive phosphorescent ligand, Pt-5,15-di (*p*-benzoato)porphyrin, as an O^2−^ sensitive probe. Then, by thiourea bonds, the RhB was covalently bonding to the amino agents in amino-quaterphenyldicarboxylate as an oxygen-independent reference. The phosphorescence/fluorescence emissions were totally independent of each other by agents sharing the same excitation energy (Figures 8C,D). The determination of oxygen content in hypoxia, normoxia, and aerated cells was realized by live-cell confocal microscopic imaging (Figures 8E–G).

Antibiotics are mainly used for infections caused by bacteria. Antibiotic resistance not only has a serious impact on human and animal health, but also can be widely spread as antibiotic wastes (Zhu et al., 2018). Due to the rapid response, highly selectivity and sensibility, the biosensors based on fluorescent MOF for antibiotic detection became popular. By adopting high-Z ions Zr^4+^ and fluorescent inorganic linkers H_3_CTTA (5'-(4-carboxyphenyl)-2′,4′,6′-trimethyl-[1,1':3′,1″-terphenyl]-4,4″-dicarboxylic acid)) and H_3_TTNA (6,6′,6''-(2,4,6-trimethylbenzene-1,3,5-triyl)tris (2-naphthoic acid)), two Zr-MOFs Zr_6_O_4_(OH)_8_(H_2_O)_4_(CTTA)_8/3_ and Zr_6_O_4_(OH)_8_(H_2_O)_4_ (TTNA)_8/3_ were constructed by Zhou’s group for sensing antibiotics effectively (Wang et al., 2016b). It was indicated that the excellent adsorption ability of these two complexes could remarkable increase the quenching efficiency and detection sensitivity for detecting and removing nitrofurazone (NZF) and nitrofurantoin (NFT) antibiotics. Recently, the author of this review designed a class of Cd(II)-based LMOFs named [Cd_2_Na(L)(BDC)_2.5_]·9H_2_O, [Cd_2_(L)(2,6-NDC)_2_]·DMF·5H_2_O and [Cd_2_(L)(BPDC)_2_]·DMF·9H_2_O (1,4-NDC^2-^ = 1,4-naphthalenedicarboxylate) by introducing chelated polyamine linker *N* ^1^-(4-(1H-1,2,4-triazole-1-yl)benzyl)-*N* ^1^-(2-aminoethyl)ethane-1,2-diamine (L). The detecting experiments indicated that these Cd-MOFs can act as potential luminescence probes for detecting antibiotics. The fluorescence intensity of these materials can be inhibited efficiently by trace amount of NZF with detection limits are about 162, 75 and 60 ppb, respectively (Zhao et al., 2017).

Biomedical imaging: The advent of biomedical imaging technologies has extremely facilitated the diagnosis of devious diseases. Imaging methods could use either fluorescence and magnetism property, that the later one is more frequent reported in reports particularly in the applications involving NMOFs in which non-ionizing non-radioactive radiation could be used to generate images. By merits of their metal connecting points or nodes, NMOFs have been proved to be outstanding contrast agents for magnetic resonance imaging (MRI), optical imaging, X-ray computed tomography (CT), and PET (Khooa et al., 1997), as shown in Table 4.

**TABLE 4:** Various NMOFs as contrast agents for biomedical imaging.

NMOFs	Active ingredient	Contrast media	Biomedical imaging technologies	Refs
Gd(BDC)_1.5_(H_2_O)_2_	Ga^3+^ ion	T1-weighted MRI	MRI	Rieter et al. (2006)
Gd-Ru	Ga^3+^ ion	T1-weighted MRI	MRI	Wang Y M et al. (2016)
Mn-NMOF	Mn^2+^ ion	T1-weighted MRI	MRI	Yang et al. (2016)
MIL-88A	Fe^3+^ ion	T2-weighted MRI	MRI	Horcajada et al. (2010)
MIL-100	Fe^3+^ ion			
Fe_3_O_4_@UiO-66	Fe_3_O_4_ nanoparticle	T2-weighted MRI	MRI	Zhao S N et al. (2016)
Zr-UiO	Zr^3+^ ion	-	CT	Kalra et al. (2004)
Hf-UiO	Hf^4+^ ion			
Au@MIL-88(Fe)	Fe^3+^ ion	T2-weighted MRI	MRI and CT	Shang et al. (2017)
Mn/Hf-IR825	Mn^2+^ ion	-	MRI and CT	Yang Y et al. (2017)
^89^Zr-UiO-66	Zr^3+^ ion	-	PET	Chen D et al. (2017)
UCNP@Fe-MIL-101-NH_2_	Fe^3+^ ion	T2-weighted MRI	Optical imaging and MRI	Li et al. (2015)
MOF@HA@ICG	indocyanine green (ICG) NIR dye Fe^3+^ ion	-	Optical imaging and PPT	Cai et al. (2017)

Magnetic Resonance Imaging (MRI). As a powerful noninvasive imaging technique, MRI contrast agents facilitate optimal tumour evaluation by positive and negative contrast media to shorten the longitudinal (T1) relaxation rates and/or reduce the transverse (T2) relaxation rates of water protons to enhance MRI.

Currently, transitional metal ion (Gd^3+^ or Mn^2+^) are generally used to construct T1 contrast agents for MRI by chelated structure to decrease severe side effects. The first example of Gd^3+^-containing NMOFs as MRI contrast agents was constructed by Lin groups, namely Gd(BDC)_1.5_(H_2_O)_2_ (Rieter et al., 2006). Gd(BDC)_1.5_(H_2_O)_2_ nanopartical shows an *r*_1_ relaxivity of 35.8 s^−1^ per mm Gd^3+^ or ≈1.6 × 107 mm^−1^ s^−1^ on a per NMOF basis. The *r*_1_ relaxivity level of Gd(BDC)_1.5_(H_2_O)_2_ is approximately an order of magnitude higher than the usually used technology Omniscan. Recently, by a hydrothermal treatment of Gd^3+^ and Ru(II)[4,4′-(COOH)_2_ bipyridyl (bpy)]_3_·Cl_2_ (L_Ru_), Yin groups acquired a kind of nanomaterial based on Gd^3+^ and L_Ru_ precursors and used for MRI, namely Gd-Ru (Wang Y M et al., 2016). SEM and TEM images indicated that Gd-Ru is in globular structures of regular shape and uniform size in a mean diameter of 138 nm. Higher MRI contrast efficiency was acquired by Gd-Ru in contrast to the commercial MRI contrast Gd–DTPA (DTPA, diethylenetriamine pentaacetic acid). Although Gd^3+^ based nanomaterial have shown good MRI ability, but the possible release of toxic Gd^3+^ ions from Gd^3+^-based nanoparticles hinders their deeper exploration for MRI. To settle the problem of toxicity of Gd-based NMOFs, Mn^2+^-based NMOFs have been extensively investigated. As an example, Lin and coworkers designed Mn-BDC and Mn-BTC NMOFs for an optional MRI contrast media by reverse-phase microemulsion techniques at room temperature and a surfactant-assisted procedure at 120°C with microwave heating (Yang et al., 2016). The *r*_1_ and *r*_2_ relaxivities of Mn-BDC NMOF were 5.5 and 80.0 mm^−1^ s^−1^ on a per Mn basis, respectively.

**FIGURE 9:**
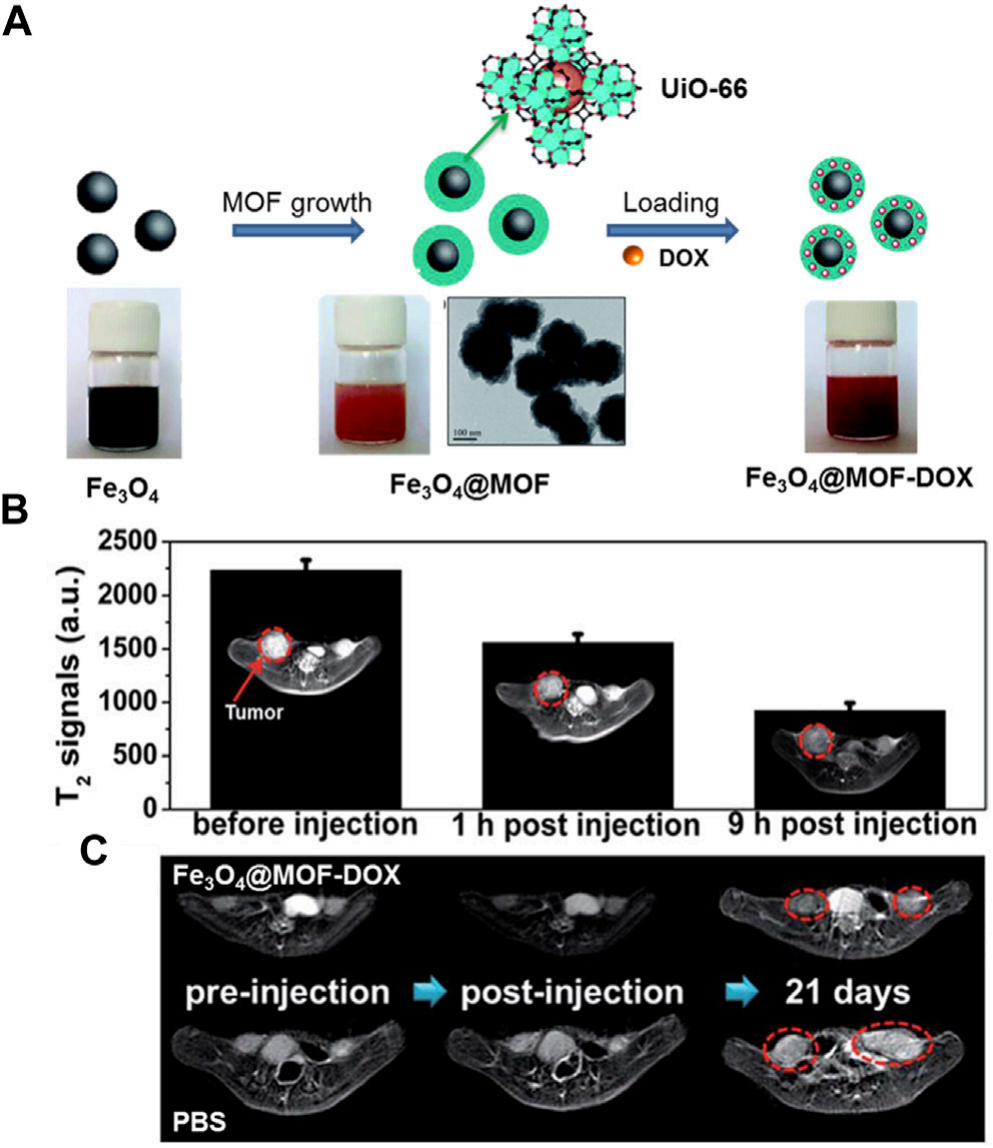
**(A)** The schematic illustration of the fabrication of Fe_3_O_4_@MOF core–shell composites. **(B)** T2-weighted MR images and T2-MR signals of tumor on HeLa-tumor bearing mice before injection, 1 and 9 h post injection of Fe_3_O_4_@UiO-66 intravenously (tumor region marked by red cycles). **(C)** MRI of HeLa-tumor bearing mice treated with PBS or Fe_3_O_4_@UiO-66-DOX collected at different time points (tumor region marked by red cycles). Source: (Zhao et al., 2016a).

The T2-weighted MRI contrast media is generally superparamagnetic iron oxides (SPIOs) which cause negative image enhancement. Not long ago, a series of ironcarboxylate NMOFs were synthesized by Horcajada and co-workers as T2-weighted contrast media (Horcajada et al., 2010). The size of these NMOFs range from 50 to 350 nm, matching known bulk phase materials of MIL-53, MIL-88A, MIL-88Bt, MIL-89, MIL-100, MIL-101-NH_2_, PEG-MIL-88A and PEG-MIL-100 exhibited high r_2_ relaxivity on a per metal basis and negative enhancement of the liver and spleen was observed after injection of MIL-88A, By incorporating Fe_3_O_4_ nanoparticles into the UiO-66, Fu groups acquired a core–shell nanomaterial Fe_3_O_4_@UiO-66 (Zhao S N et al., 2016), as shown in Figure 9A. Drug DOX was used to assessment the drug loading ability of the Fe_3_O_4_@UiO-66 and incorporated into the framework of Fe_3_O_4_@UiO-66 to obtain Fe_3_O_4_@UiO-66-DOX. The generated Fe_3_O_4_@UiO-66-DOX can not only act as nanoplatform for simultaneous drug delivery, but also can serve as a contrast media for T2-weighted MRI (Figure 9B). Moreover, Fe_3_O_4_@UiO-66-DOX could effectively inhibit the growth of tumor, and the MRI of HeLa-tumor bearing mice injected with Fe_3_O_4_@UiO-66-DOX revealed obvious darkening effect in the tumor area (Figure 9C).

X-Ray Computed Tomography Imaging (CT). CT imaging is a noninvasive clinical diagnosis technique for 3D visualization of internal structures of a scanned object by X-ray attenuation. Contrast agents with high-Z ions, such as small molecular contrast media including iodinated aromatic molecules and barium sulfate, normally exhibit strong X-ray attenuation (Kalra et al., 2004). For the high-Z elements can be facile merged into the frameworks of NMOFs with extremely high payloads, NMOFs have been a prospective contrast platform for CT. At present, lots of NMOFs with high-Z ions have been obtained and applied as possible CT contrast media. Two NMOFs (Zr-UiO and Hf-UiO) with high loadings of Zr (37 wt%) and Hf (57 wt%) were developed by Lin and co-workers and finally their potential applications as CT contrast agents were demonstrated (Dekrafft et al., 2012). The Zr and Hf NMOFs with corresponding atomic number of 40, 72 respectively making those potential candidates as CT contrast agents. Due to strong X-ray absorbance, Au nanoparticles also could be used as contrast media for CT imaging. Tian groups described an Au nanorod-incorporated core–shell material named Au@MIL-88(Fe), could serve as a multi-mode diagnostic system to provide high resolution image in either MRI or CT imaging by microemulsion method in DMF (Shang et al., 2017). The as-prepared Au@MIL-88(A) nanomaterials in a well-defined star-like morphology structure had high monodispersity and homogeneity. Both CT and MRI induced by Au@MIL-88(Fe) contrast agents showed distinct tumor boundaries with high penetration depth and spatial resolution. Liu groups used IR825 as organic linker to coordinate with Mn^2+^ and Hf^4+^ ions for preparing core-shell NMOFs to act as multi-mode imaging agents (Yang Y et al., 2017). In the NMOF, Mn^2+^ ion are acted as a MRI contrast media; high-Z element Hf^4+^ ion can enhance CT signal and radio-sensitization, and the ligand IR825, is used as a photothermal agent due to the strong NIR absorption.

Positron Emission Tomography (PET). PET is X-ray functional imaging method with superior detection sensitivity (as low as picomolar scope) and stronger signal penetration. In PET, the radionuclides emitted by positrons gather on the target organ, producing a pair of gamma ray photons that can be trapped by sensitive detector panels. Hong and co-workers have done outstanding work on exploring nanomaterial for PET imaging. Recently, they obtained ^89^Zr-containing NMOF for PET (Chen X et al., 2017). Besides the ^89^Zr was loading into the framework of UiO-66, the surfaces of the nanomaterial were functionalized by pyrene-derived PEG further. PET scans traced the organ distribution of ^89^Zr-UiO-66/Py−PGA-PEG-F3 *in vivo* and detected an 8.2 ± 0.3% total injected dose per Gram of tumor after intravenous injection for 2 h.

Optical Imaging. Optical imaging, a kind of fluorescence technique, is widespread in biomedicine. Generally excited dye molecules by visible light in organism and produce luminescence. Highly sensitive and minimally invasive make the optical imaging widely used in clinic.

**FIGURE 10:**
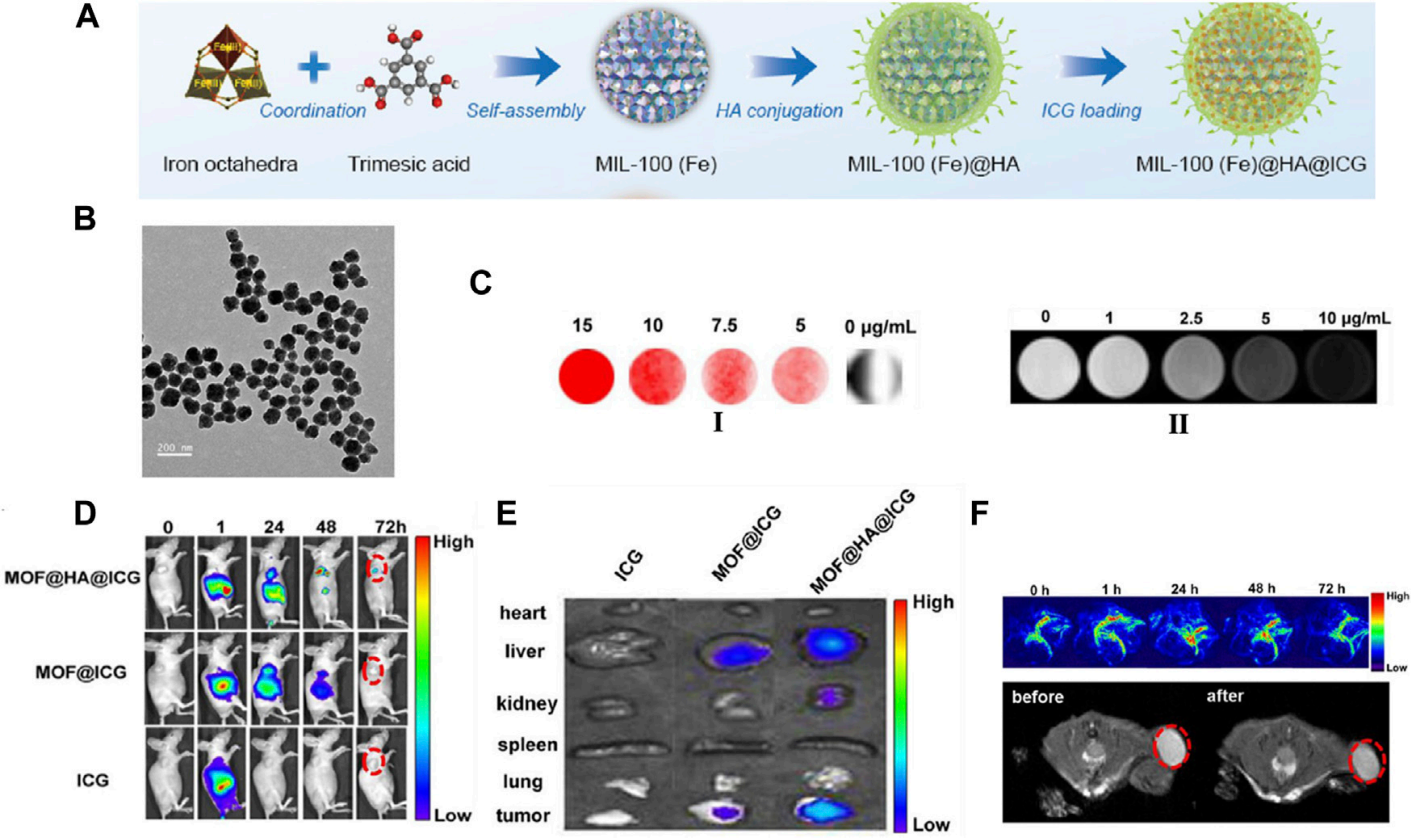
**(A)** Schematic description of the synthesis process of HA conjugated, ICG loading of MIL-100(Fe) nanomaterial. **(B)** TEM images of the MOF@HA@ICG NPs. **(C)** FL images (I) and T2-weighted MR images (II) of cells treated with MOF@HA@ICG solutions. **(D)** FL images of MCF-7 tumor-bearing mice injected with free ICG, MOF@ICG, or MOF@HA@ICG solutions (each with 170 μg/ml of ICG; the tumors are highlighted by red circles). **(E)** *In vitro* FL images of organs (heart, liver, kidney, spleen, and lung) and tumors. **(F)** *In vivo* PA images and T2-weighted MR images with MOF@HA@ICG treatment. The tumors are highlighted by red circles. Source: (Li et al., 2015).

Recently, by incorporated Yb^3+^ and phenylene-derived photosensitizers, Foucault-Collet and co-workers created unique near-infrared-emitting Yb^3+^-containing NMOFs (Foucault-Collet et al., 2013). Accompanied by being engulfed by cells and reserved in cytoplasm, Yb^3+^-based NMOFs emit the number of photons per unit volume that higher enough to realize the real-time imaging of HeLa and NIH 3T3 cells. Li and co-workers constructed core-shell nanomaterial with integrate of single NaYF_4_: Yb, Er upconversion nanoparticles (UCNPs) into Fe-MIL-101-NH_2_ to realize luminescent/magnetic dual-mode imaging (Li et al., 2015). The UCNP@Fe-MIL-101-NH_2_ was further surface-functionalized with PEG and PEG-folic acid (FA) to improve the biocompatibility. A significant KB tumor contrast could be observed after intravenous injection for 24 h *in vivo* optical imaging. Furthermore, the dyes with optical activity were also inserted within the skeleton of NMOFs either by post synthetically or as an object species to realize the optical imaging. For example, a case of hyaluronic acid (HA) incorporated MIL-100 (Fe) NMOFs were developed by Cai and co-workers (Figures 10A,B). By inserting the indocyanine green (ICG) NIR dye into the NMOFs to realize image-induced PPT ^[94]^, this material exhibited a strong load capacity of ICG (40 wt%), high optical stability, and strong NIR absorbance (Figures 10C,D). FL images of the *in vivo* and *in vitro* certificated that MOF@HA@ICG had excellent uptake capacity of CD44-positive MCF-7 cells (Figures 10E,F).

## Conclusion and Outlook

In the last few years, substantial improvement has been achieved in applying NMOFs for drug delivery, cancer therapy and biomedical sensing and imaging. Various NMOFs-based nanomedicine have been intelligently designed and proved to be effective and powerful for clinic both *in vivo* and *in vitro*. In this review, we have comprehensively discussed the update achievements of NMOFs in clinic from a broader vision including applying NMOFs as nanocarriers, anti-cancer therapeutic agents, biosensors and molecular imaging probes as well as well-established synthetic methods for the strengthening their functional capabilities and extending their potential nanomedicine applications. Furthermore, some key issues of NMOFs including controlled synthesis, surface modification, toxicity, biocompatibility, stability, and therapeutic/imaging efficacy before the biomedical applications of NMOFs also are addressed in this review. Despite the sustainable growing interest and impressive accomplishment has been obtained in this area, the development of NMOFs as drug delivery, cancer therapy and biomedical sensing and imaging agents is still in its infancy contrasted to other nanocarriers.

Generally, it can be seen that NMOFs hold obvious superiority compared with other nanoformulations in nanomedicine applications because of their intrinsic characteristics including highly porous and large channels, structural adjustability/multifunctionality, as well as low toxicity and biocompatibility. Nevertheless, such nanomedical system has boundedness, such as complicated hierarchy for targeted drug delivery without eliminated or undergoes degradation, pure efficiency of phototherapy and photodynamic therapy, difficult fabricating for sensor device and so on. Consequently, there is still defiance in this field and deeper and constant exploration is needed. From a viewpoint of synthetic strategy, one of the biggest challenges is to explore new synthetic methods for the fabrication of smaller-size NMOFs on account of smaller NMOFs has better applicability than larger ones. Still, the smaller NMOFs are better for the integration of biomolecules, particularly in FRET. From the point view of applications, further efforts should be paid to prepare versatile biocompatible and low-toxicity NMOFs. Besides that, chemical instability of NMOFs is essential. Additionally, although lots of researches have proved the short-term safety of NMOFs *in vitro* or *in vivo* studies, the long-term chronic toxicity effects, pathway, and clearance behavior of NMOFs are rarely explored.

In brief, multi-functional NMOFs engineering for nanomedicine is a newly burgeoning field. There is still a large innovative research space in this exciting area. For better applied the NMOFs in disease diagnosis and treatment of clinic application, additional breakthroughs needed to acquire for solving all obstacles mentioned above in the near future.
